# Radiosynthesis, structural identification and in vitro tissue binding study of [^18^F]FNA-*S*-ACooP, a novel radiopeptide for targeted PET imaging of fatty acid binding protein 3

**DOI:** 10.1186/s41181-024-00245-3

**Published:** 2024-02-23

**Authors:** Pyry Dillemuth, Tuomas Karskela, Abiodun Ayo, Jesse Ponkamo, Jonne Kunnas, Johan Rajander, Olli Tynninen, Anne Roivainen, Pirjo Laakkonen, Anu J. Airaksinen, Xiang-Guo Li

**Affiliations:** 1 Turku PET Centre and Department of Chemistry, University of Turku, Turku, Finland; 2 Translational Cancer Medicine Research Program, Faculty of Medicine, University of Helsinki, Helsinki, Finland; 3 Pharmaceutical Sciences Laboratory, Faculty of Sciences and Engineering, Åbo Akademi University, Turku, Finland; 4 Accelerator Laboratory, Åbo Akademi University, Turku, Finland; 5 Department of Pathology, Helsinki University Hospital and University of Helsinki, Helsinki, Finland; 6 Turku PET Centre, University of Turku and Turku University Hospital, Kiinamyllynkatu 4-8, 20520 Turku, Finland; 7 Turku Center for Disease Modeling, University of Turku, Turku, Finland; 8 InFLAMES Research Flagship, University of Turku, Turku, Finland; 9 Laboratory Animal Centre, HiLIFE University of Helsinki, Helsinki, Finland; 10 iCAN Flagship Program, University of Helsinki, Helsinki, Finland

**Keywords:** ACooP, Peptide, Autoradiography, Fatty acid binding protein 3, Fluorine-18, Peptide radiolabeling, PET, *S*-acylation

## Abstract

**Background:**

Fatty acid binding protein 3 (FABP3) is a target with clinical relevance and the peptide ligand ACooP has been identified for FABP3 targeting. ACooP is a linear decapeptide containing a free amino and thiol group, which provides opportunities for conjugation. This work is to develop methods for radiolabeling of ACooP with fluorine-18 (^18^F) for positron emission tomography (PET) applications, and evaluate the binding of the radiolabeled ACooP in human tumor tissue sections with high FABP3 expression.

**Results:**

The prosthetic compound 6-[^18^F]fluoronicotinic acid 4-nitrophenyl ester was conveniently prepared with an on-resin ^18^F-fluorination in 29.9% radiochemical yield and 96.6% radiochemical purity. Interestingly, 6-[^18^F]fluoronicotinic acid 4-nitrophenyl ester conjugated to ACooP exclusively by *S*-acylation instead of the expected *N*-acylation, and the chemical identity of the product [^18^F]FNA-*S*-ACooP was confirmed. In the in vitro binding experiments, [^18^F]FNA-*S*-ACooP exhibited heterogeneous and high focal binding in malignant tissue sections, where we also observed abundant FABP3 positivity by immunofluorescence staining. Blocking study further confirmed the [^18^F]FNA-*S*-ACooP binding specificity.

**Conclusions:**

FABP3 targeted ACooP peptide was successfully radiolabeled by *S*-acylation using 6-[^18^F]fluoronicotinic acid 4-nitrophenyl ester as the prosthetic compound. The tissue binding and blocking studies together with anti-FABP3 immunostaining confirmed [^18^F]FNA-*S*-ACooP binding specificity. Further preclinical studies of [^18^F]FNA-*S*-ACooP are warranted.

**Supplementary Information:**

The online version contains supplementary material available at 10.1186/s41181-024-00245-3.

## Background

Peptides are favorable vehicles for the delivery of radionuclides for nuclear imaging and radiotherapy. Among the 14 radiolabeled organic compounds approved by the U.S. Food and Drug Administration for clinical imaging with positron emission tomography (PET), six are peptides or peptide analogues (http://www.radiopharmaceuticals.info/radiopharmaceuticals.html). In the international research community, much research endeavors have been devoted to the development of novel peptide-based radiopharmaceuticals and methodologies for peptide radiolabeling (Krecisz et al. 2021). We have been developing several new peptides and their radiolabeling methods for PET imaging of inflammation and cancer, targeting different types of biological targets (Käkelä et al. 2018; Li et al. 2013). Recently, using an in vivo phage display technique, Laakkonen and co-workers identified a brain tumor-homing peptide (named ACooP, sequence H-ACGLSGLGVA-NH_2_) that targets the fatty acid binding protein 3 (FABP3, also known as mammary-derived growth inhibitor or heart-type fatty acid-binding protein) (Hyvönen et al. 2014). The targeting affinity and specificity of ACooP and its several variants have been studied in detail in different experimental settings (Feng et al 2015; Lico et al. 2021). FABP3 plays a significant role in cancer and neurodegenerative diseases (Kawahata et al. 2019; McKillop et al. 2019). For example, in a study involving 1331 patients with breast cancer, 94.3% of tumor samples were FABP3-positive, and this was associated with better 10-year survival rate (Nevo et al. 2010). As another example, FABP3 expression was colocalized with α-synuclein aggregates in the human brain tissue sections with synucleinopathies (Oizumi et al. 2021). Furthermore, FABP3 expression has been linked to several other diseases, as detailed in a recent review (Mallick et al. 2021). Given the growing body of clinical evidence implicating FABP3 expression in human diseases, we hypothesize that FABP3 could be a potential target for PET imaging. Accordingly, we set out to radiolabel ACooP peptide with fluorine-18 (^18^F) for FABP3-targeted PET imaging applications.

**Fig. 1:**
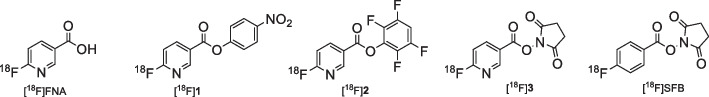
Examples of ^18^F-prosthetic groups for *N*-acylation of biomolecules

[^18^F]Fluoronicotinic acid ([^18^F]FNA, Fig. 1) is one of the favorable ^18^F-prosthetic groups for radiolabeling of biomolecules including peptides and antibody fragments (Basuli et al. 2016, 2020; Haskali et al. 2020; Keam 2021; Zhou et al. 2019). The clinical utility of [^18^F]FNA-conjugates has been demonstrated by the success of Piflufolastat F-18 used in PET imaging of prostate-specific membrane antigen (PSMA)–positive prostate cancer lesions and metastases (Keam 2021). To conjugate [^18^F]FNA with amino group functionalized molecules with *N*-acylation reaction, activated esters [^18^F]**1**–**3** of [^18^F]FNA have been used (Fig. 1) (Haskali et al. 2020; Basuli et al. 2016, 2020). In general, the radiosynthesis of [^18^F]**1**–**3** is more straightforward compared to one of the earlier generation *N*-acylation agents, *N*-succinimidyl-4-[^18^F]fluorobenzoate ([^18^F]SFB, Fig. 1) (Vaidyanathan and Zalutsky 2006). For example, [^18^F]**2** and [^18^F]**3** have been conveniently prepared by on-resin ^18^F-fluorination at room temperature (r.t.), simply by passing the precursor solutions through [^18^F]fluoride-bound anion-exchange cartridges (Basuli et al. 2016, 2020).

**Fig. 2:**
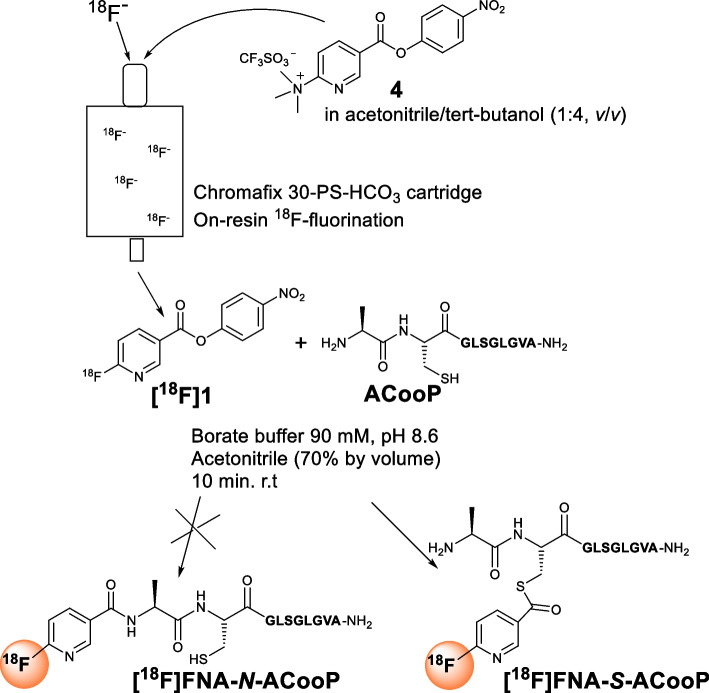
Radiosynthesis of [^18^F]FNA-*S*-ACooP

**Fig. 3:**
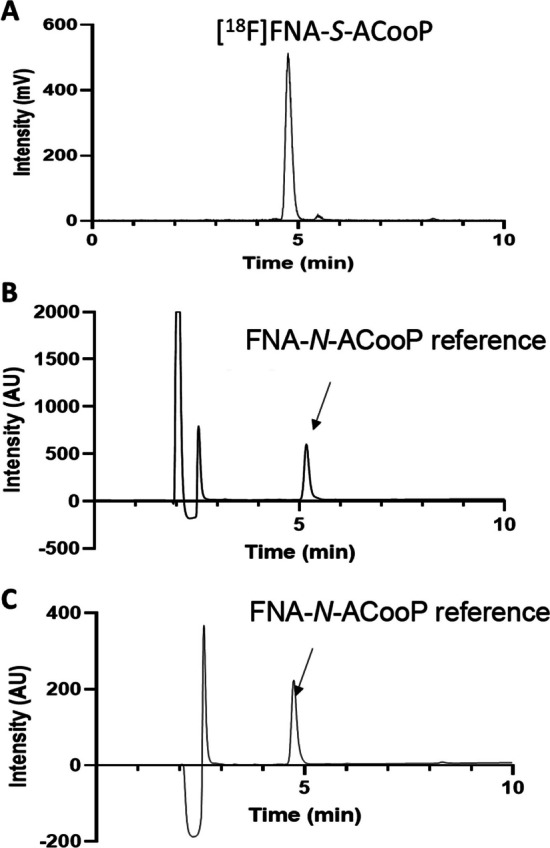
Identification and quality control of [^18^F]FNA-*S*-ACooP. HPLC chromatograms of [^18^F]FNA-*S*-ACooP under radioactivity detection (**A**) and reference compound FNA-*N*-ACooP (**B**) and [^18^F]FNA-*S*-ACooP (**C**) under UV detection

**Fig. 4:**
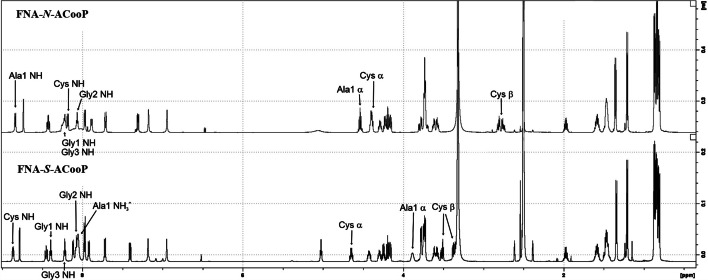
^1^H NMR spectra of FNA-*N*-ACooP and FNA-*S*-ACooP

In this work, we chose to use [^18^F]**1** as the prosthetic compound for radiolabeling of ACooP peptide (Fig. 2), as its precursor compound *N*,*N*,*N*-trimethyl-5-((4-nitrophenoxy)carbonyl)pyridine-2-aminium triflate (**4**) is commercially available and [^18^F]**1** is not volatile, which is important from radiation safety point of view. Here, we demonstrate that it is feasible to use on-resin ^18^F-fluorination to prepare [^18^F]**1**, and [^18^F]**1** has high chemoselectivity towards *S*-acylation instead of the expected *N*-acylation in the case of ACooP peptide conjugation. Furthermore, we carried out structural characterization in details to confirm the chemical identity of the radiolabeled product [^18^F]FNA-*S*-ACooP (Figs. 3, 4) and studied its in vitro binding using brain metastasis tissue sections from a patient with lung cancer.

## Methods

### Materials and general methods

Precursor compound *N,N,N*-trimethyl-5-((4-nitrophenoxy)carbonyl)pyridin-2-aminium trifluoromethanesulfonate (**4**) was purchased from R&S Chemicals (Kannapolis, NC, USA). ACooP and FNA-*N*-ACooP peptides was purchased from United Biosystems (Herndon, VA, USA). Polyclonal rabbit anti-human FABP3 antibody (sc-15974-R) was purchased from Santa Cruz (Dallas, TX, USA). Monoclonal mouse anti-human PECAM/CD31 and endothelial cell antibody (clone JC70A) was purchased from Dako (Glostrup, Denmark). Alexa Fluor (AF) conjugated secondary antibodies, including goat anti-mouse AF488 and goat anti-rabbit AF647, were purchased from Thermo Fisher (Life Technologies, Waltham, MA, USA). Nuclear magnetic resonance (NMR) spectra were recorded using a Bruker Avance III 600 MHz equipped with a liquid nitrogen cooled Prodigy TCI probe or a Bruker Avance III 500 MHz equipped with a liquid nitrogen cooled Prodigy BBO probe. Chemical shifts of ^1^H NMR were reported relative to the solvent residual proton signal of dimethyl sulfoxide (DMSO-*d*6: δ = 2.50 ppm). Chemical shifts of ^13^C NMR were reported relative to the solvent signal (DMSO-*d*6: δ = 39.52 ppm). Methods for high-resolution mass spectrometry (HRMS) and liquid chromatography-mass spectrometry (LC–MS) were described in the Additional file 1.

### Synthesis of fluoronicotinic acid 4-nitrophenyl ester (1)

**1** was prepared according to a published method with modifications (Haskali 2020). 6-Fluoronicotinic acid (2.00 mmol, 282 mg), 4-nitrophenol (2.00 mmol, 278 mg), 4-dimethylaminopyridine (DMAP, 0.12 mmol, 15 mg) and a magnetic stir bar were charged into a 25 mL round bottom flask. The contents of the round bottom flask were dissolved with 5 mL of dry acetonitrile and allowed to stir for 40 min at r.t. Once the allotted time had elapsed, 1-ethyl-3-(3-dimethylaminopropyl)carbodiimide (EDC, 2.40 mmol, 372 mg) was added piecemeal to the solution, after which a thick white slurry formed. The reaction was monitored by thin-layer chromatography (TLC) using a mixture of solvents hexane:ethylacetate (3:1, *v*/*v*) as an eluent. The reaction mixture was concentrated *in vacuo* and the resultant solid was dissolved with dichloromethane and charged into a separatory funnel, which was then washed 3 times with 0.1 M HCl (10 mL washes). The dichloromethane fraction was then washed with saturated sodium chloride solution and dried over anhydrous magnesium sulfate. Magnesium sulfate was removed by filtering through filter paper and concentrating the crude product *in vacuo* to a white solid. The crude product was purified by silica gel column chromatography with a solution of hexane:ethylacetate (3:1, *v*/*v*) as eluent, and the resulting fractions were collected and concentrated to dryness *in vacuo.* The final product was obtained as a white solid (255 mg, 0.97 mmol, 49% yield). ^1^H NMR (500 MHz, CDCl_3_, Additional file 1: Fig. S1) δ 9.09 (d, J = 2.5 Hz, 1H), 8.58 (m, 1 H), 8.36 (d, J = 9.2 Hz, 2H), 7.46 (d, J = 9.2 Hz, 2H), 7.15 (dd, J = 2.9, 8.6 Hz, 1H). ^13^C NMR (101 MHz, CDCl_3_) δ 166.46 (d, J = 248.3 Hz) 161.96, 154.95, 151.24 (d, J = 17.1 Hz), 145.75, 143.23 (d, J = 9.6 Hz), 125.43, 123.13 (d, J = 4.5 Hz), 122.53, 110.17 (d, J = 37.5 Hz). ^19^F NMR (471 MHz, CDCl_3_) δ -58.77 (d, J = 6.2 Hz). HRMS: [M + H]^+^ Calculated: 263.0468, observed m/z = 263.0462.

### Preparation of FNA-*S*-ACooP

A 1.5 mL Eppendorf vial was charged with 33 µL borate buffer (300 mM, pH 8.6), 100 µL 6.4 mM ACooP solution in water (TraceSELECT Honeywell), 33 µL 60 mM FNA in acetonitrile and 200 µL acetonitrile, which was agitated, and then allowed to react at r.t. for 8–10 min. The reaction mixture was then purified by a semi-preparative high-performance liquid chromatography (HPLC) C18 column (Jupiter Proteo, 250 × 10 mm, 4 µm, 90 Å; Phenomenex, Torrance, CA, USA) at a flow rate of 4 mL/min. Solvent A was 0.1% trifluoroacetic acid (TFA) in water and solvent B was 0.1% TFA in acetonitrile. The HPLC elution gradient was during 0–14.8 min from 20% B to 50% B, and 14.8–16.0 min 50% B to 80% B. The collected fraction appeared at a retention time of 8.05 min and was then lyophilized. The above procedure was repeated twice and the fractions containing the product were combined and concentrated in dryness. The obtained product (1.1 mg, 1.14 µmol, yield 89%) was characterized with MS, one-dimensional (1D) and two-dimensional (2D) NMR (Additional file 1: Fig. S2–8). 1D NMR analysis data were described as follow. δ^1^H(600 MHz; DMSO-*d*6) 0.81 (3 H, d, Me(Val)), 0.83 (3 H, d, Me(Leu)), 0.84 (3 H, d, Me(Leu)), 0.85 (3 H, d, Me(Val)), 0.87 (6 H, d, Me(Leu)), 1.21 (3 H, d, Me(Ala2)), 1.34 (3 H, br, Me(Ala1)), 1.46 (4 H, m, CH_2_(Leu1,2β)), 1.59 (2 H, m, CH(Leuγ)), 1.97 (1 H, m, CH(Valβ)), 3.37 (1 H, dd, CH_2_(Cysβ)), 3.51 (1 H, dd, CH_2_(Cysβ)), 3.57 (1 H, dd, CH_2_(Serβ)), 3.61 (1 H, dd, CH_2_(Serβ)), 3.73 (2 H, d, CH_2_(Glyα)), 3.75 (2 H, d, CH_2_(Glyα)), 3.78 (2 H, d, CH_2_(Glyα)), 3.89 (1 H, br, CH(Ala1α)), 4.16 (1 H, dd, CH(Valα)), 4.19 (1 H, m, CH(Ala2α)), 4.24 (1 H, m, CH(Serα)), 4.30 (1 H, m, CH(Leu2α)), 4.43 (1 H, m, CH(Leu1α)), 4.65 (1 H, m, CH(Cysα)), 5.02 (1 H, t, CH(SerOH)), 6.94 (1 H, s, CONH_2_(Ala2)), 7.18 (1 H, s, CONH_2_(Ala2)), 7.40 (1 H, dd, 5-H(FPy)), 7.72 (1 H, d, NH(Val)), 7.92 (1 H, d, NH(Leu2)), 7.97 (2 H, d, NH(Ala2), NH(Leu1)), 8.05 (2 H, br, NH(Ala1)), 8.07 (1 H, t, NH(Gly2)), 8.12 (1 H, d, NH(Ser)), 8.22 (1 H, t, NH(Gly3)), 8.39 (1 H, t, NH(Gly1)), 8.44 (1 H, dt, 4-H(FPy)), 8.78 (1 H, d, 2-H(FPy)), 8.86 (1 H, d, NH(Cys)). δ^13^C(125 MHz; DMSO-*d*6) 17.11 Me(Ala1), 17.88 Me(Val), 18.17 Me(Ala2), 19.14 Me(Val), 21.49 Me(Leu), 21.55 Me(Leu), 23.00 Me(Leu), 23.17 Me(Leu), 24.07 Cγ(Leu1, Leu2), 30.53 Cβ(Val), 30.66 Cβ(Cys), 40.83 Cβ(Leu), 40.96 Cβ(Leu), 41.99 Cα(Gly), 42.03 Cα(Gly), 42.14 Cα(Gly), 47.94 Cα(Ala2), 48.16 Cα(Ala1), 50.77 Cα(Leu2), 51.01 Cα(Leu1), 51.66 Cα(Cys), 55.26 Cα(Ser), 57.49 Cα(Val), 61.65 Cβ(Ser), 110.55 (d, 5-C(FPy), 130.87 (d, 3-C(FPy), 141.08 (d, 4-C(FPy), 147.20 (d, 2-C(FPy), 165.34 (d, 6-C(FPy), 168.01 (CO(Gly)), 168.64 (CO(Gly)), 168.78 (CO(Gly)), 169.07 (CO(Cys)), 169.68 (CO(Ala1)), 170.30 (CO(Val)), 170.32 (CO(Ser)), 172.20 (CO(Leu2)), 172.30 (CO(Leu1)), 174.05 (CO(Ala2)), 188.07 (CO(FPy)). HRMS (ESI) m/z: [M + H]^+^ Calculated for C_41_H_66_N_12_O_12_FS 969.4622; found 969.4626.

### Structural characterization of FNA-*N*-ACooP

To facilitate the structure identification of FNA-*S*-ACooP, reference compound FNA-*N*-ACooP was purchased as a custom synthesis and characterized with MS, 1D and 2D NMR (Additional file 1: Fig. S9–15). 1D NMR analysis data were described as follow. δ^1^H(600 MHz; DMSO-*d*6) 0.81 (3 H, d, Me(Val)), 0.82 (3 H, d, Me(Leu)), 0.83 (3 H, d, Me(Leu)), 0.85 (3 H, d, Me(Val)), 0.87 (6 H, d, Me(Leu)), 1.2β (3 H, d, Me(Ala2)), 1.35 (3 H, d, Me(Ala1)), 1.46 (4 H, m, CH_2_(Leu1,2β)), 1.58 (2 H, m, CH(Leuγ)), 1.97 (1 H, m, CH(Valβ)), 2.75 (1 H, dd, CH_2_(Cysβ)), 2.81 (1 H, dd, CH_2_(Cysβ)), 3.57 (1 H, dd, CH_2_(Serβ)), 3.62 (1 H, dd, CH_2_(Serβ)), 3.69–3.81 (6 H, m, CH_2_(Glyα)), 4.16 (1 H, dd, CH(Valα)), 4.19 (1 H, m, CH(Ala2α)), 4.23 (1 H, m, CH(Serα)), 4.29 (1 H, m, CH(Leuα)), 4.39 (1 H, m, CH(Cysα)), 4.41 (1 H, m, CH(Leuα)), 4.54 (1 H, m, CH(Ala1α)), 5.05 (1 H, br, OH(Ser)), 6.94 (1 H, s, CONH_2_(Ala2)), 7.17 (1 H, s, CONH_2_(Ala2)), 7.31 (1 H, dd, 5-H(FPy)), 7.71 (1 H, d, NH(Val)), 7.88 (1 H, d, NH(Leu)), 7.97 (1 H, d, NH(Ala2)), 8.02 (1 H, br, NH(Leu)), 8.06 (1 H, t, NH(Gly2)), 8.10 (1 H, br, NH(Ser)), 8.17 (1 H, d, NH(Cys)), 8.22 (2 H, m, NH(Gly1, Gly3)), 8.42 (1 H, dt, 4-H(FPy)), 8.73 (1 H, d, 2-H(FPy)), 8.84 (1 H, d, NH(Ala1)). δ^13^C(125 MHz; DMSO-*d*6) 17.53 Me(Ala1), 17.90 Me(Val), 18.16 Me(Ala2), 19.15 Me(Val), 21.51 Me(Leu), 21.56 Me(Leu), 23.00 Me(Leu), 23.15 Me(Leu), 24.05 Cγ(Leu), 24.07 Cγ(Leu), 26.14 Cβ(Cys), 30.53 Cβ(Val), 40.79 Cβ(Leu), 40.85 Cβ(Leu), 41.98 Cα(Gly), 42.06 Cα(Gly), 42.18 Cα(Gly), 47.96 Cα(Ala2), 49.14 Cα(Ala1), 50.86 Cα(Leu2), 51.04 Cα(Leu1), 55.08 Cα(Cys), 55.31 Cα(Ser), 57.52 Cα(Val), 61.63 Cβ(Ser), 109.33 (d, 5-C(FPy), 128.34 (d, 3-C(FPy), 141.73 (d, 4-C(FPy), 147.69 (d, 2-C(FPy)), 163.76 CO(FPy), 164.25 (d, 6-C(FPy)), 168.47 (CO(Gly)), 168.67 (CO(Gly)), 168.80 (CO(Gly)), 169.93 (CO(Cys)), 170.33 (CO(Val)), 170.36 (COβSer)), 172.13 (CO(Leu2)), 172.28 (CO(Leu1)), 172.30 (CO(Ala1)), 174.06 (CO(Ala2)). HRMS (ESI) m/z: [M + H]^+^ Calculated for C_41_H_66_N_12_O_12_FS 969.4622; found 969.4619.

### Radiosynthesis of [^18^F]FNA-***S***-ACooP

Prosthetic compound [^18^F]**1** was prepared with an on-resin ^18^F-fluorination method as previously reported (Fig. 2) (Basuli et al. 2016). Accordingly, [^18^F]fluoride (3.5–10.0 GBq) was produced in-house by ^18^O(p,n)^18^F nuclear reaction and extracted onto a Chromafix 30-PS-HCO_3_ anion-exchange cartridge (Macherey-Nagel GmbH & Co. KG, Düren, Germany). The cartridge was flushed with anhydrous acetonitrile and dried with N_2_ gas for 3 min. [^18^F]**1** was synthesized by passing through the cartridge with a solution of compound **4** (8 mg, 17.74 µmol) and 1,4-diazabicyclo[2.2.2]octane (DABCO, 14 mg, 0.13 mmol) in acetonitrile:tert-butanol (1:4, *v*/*v*, 1.2 mL). The ^18^F-fluorination efficiency tests were performed using different amounts of precursor **4** in the presence or absence of DABCO (Additional file 1: Table S1). [^18^F]**1** was purified using HPLC on a reversed-phase C18 column (Jupiter Proteo, 250 × 10 mm, 5 µm, 90 Å; Phenomenex, Torrance, CA, USA) at a flow rate of 4 mL/min. Solvent A was 0.1% TFA in water and solvent B was 0.1% TFA in acetonitrile. The HPLC elution gradient was during 0–15 min from 45 to 70% B. The HPLC fraction containing [^18^F]**1** was diluted with 25 mL water and two Sep-Pak tC18 Plus Light Cartridges (tC18; Waters, Milford, MA, USA) were connected to extract [^18^F]**1**. Subsequently, [^18^F]**1** was eluted from tC18 cartridges with 0.8 mL acetonitrile into a reaction vial to which a solution of ACooP (5 mg, 5.91 µmol) in 350 μL borate buffer (300 mM, pH 8.6) was added. The reaction mixture was kept at r.t. for 10 min and subjected to HPLC purification on a reversed-phase C18 column (Jupiter Proteo, 250 × 10 mm, 5 µm, 90 Å; Phenomenex) at a flow rate of 4 mL/min. Solvent A was 0.1% TFA in water and solvent B was 0.1% TFA in acetonitrile. The HPLC elution gradient was 0% B during 0–5 min and 5–25 min from 0 to 30% B. The HPLC fraction containing [^18^F]FNA-*S*-ACooP was diluted with 25 mL water and [^18^F]FNA-*S*-ACooP was extracted onto a tC18 cartridge. [^18^F]FNA-*S*-ACooP was eluted out with a mixture of 250 μL ethanol, 200 μL water (TraceSELECT, Honeywell, Charlotte, NC, USA) and 50 μL of ascorbic acid (150 mM in water) into the end product vial, which was prefilled with 1.4 mL phosphate-buffered saline (PBS) containing ascorbic acid (14 mM). The peptide conjugation efficiency tests were performed using ACooP at different concentrations in the reaction mixture (Additional file 1: Fig. S16).

Radiochemical purity and identity of [^18^F]FNA-*S*-ACooP were analyzed by HPLC. Accordingly, a mixture of [^18^F]FNA-*S*-ACooP (0.5–1.0 MBq) and 10 nmol of cold reference FNA-*S*-ACooP in 50 μL of PBS containing ascorbic acid (14 mM) was injected into a reversed-phase C18 column (Jupiter Proteo, 250 × 4.6 mm, 5 µm, 90 Å; Phenomenex) at a flow rate of 1 mL/min under radioactivity detection and ultraviolet (UV, wavelength 220 nm) detection. Solvent A was 0.1% TFA in water and solvent B was 0.1% TFA in acetonitrile. The HPLC elution gradient was from 27 to 42% B during 0–10 min. The decay-corrected radiochemical yield was 29.9% ± 2.3 (*n* = 4) and radiochemical purity was 96.6% ± 2.3. The molar activity was 36.2 ± 22.0 MBq/nmol at the end of synthesis. The total synthesis time was 181.5 ± 6.9 min from the end of bombardment. The shelf-life of [^18^F]FNA-*S*-ACooP in the injectable formulation (PBS with 14 mM ascorbic acid) was investigated by taking samples at different time points up to 4 h for HPLC analysis as described above.

### Log***D***_7.4_ measurements

The distribution coefficient Log*D*_7.4_ was measured by topping up 5 kBq of the tracer [^18^F]FNA-*S*-ACooP with PBS (pH 7.4) to 600 µL and adding 600 µL of 1-octanol. The solution was mixed thoroughly for 3 min and then centrifuged for 3 min at 12,000 × *g* to separate the layers. Aliquots of 400 µL each were taken from both layers and the radioactivity was measured using a gamma counter. The tests were performed in triplicate. Log*D*_7.4_ of [^18^F]FNA-*S*-ACooP was calculated as follows with radioactivity decay correction: ${\text{Log}}D = \log 10\frac{counts\;in\;the\;octanol\;phase}{{counts\;in\;the\;PBS\;phase}}$. Log*D*_7.4_ of [^18^F]FNA-*S*-ACooP was − 0.55 ± 0.01 (*n* = 3).

### In vitro tissue binding and autoradiography

Cryosections (20 µm thickness, *n* = 4) of a brain metastasis sample from a patient with lung cancer were defrosted at 4 °C for 15 min followed by r.t. for 15 min and incubated in PBS (pH 7.4) at r.t. for 15 min. The slides were then incubated in PBS containing [^18^F]FNA-*S*-ACooP at a radioactivity concentration of 0.018 MBq/mL for 45 min at r.t., rinsed twice with 4 °C PBS for 2 min, and dipped once with water at 4 °C. Slides were dried with gentle air flow and exposed to a BAS-TR2025 phosphor imaging plate (Fujifilm, Tokyo, Japan). After an exposure time of 18 h, the imaging plates were scanned with a BAS-5000 scanner (Fujifilm, Tokyo, Japan), and the autoradiography images were viewed with Carimas 2.10 software (Turku PET Centre, Turku, Finland, www.turkupetcentre.fi/carimas/). The blocking experiments were performed with similar protocols as described above, except that the tissue cryosections were first incubated with molar excess of ACooP (5 μM) for 15 min before addition of [^18^F]FNA-*S*-ACooP (0.5 nM). The radioactivity binding in the tissue samples was quantified as photostimulated luminescence units per square millimeter (PSL/mm^2^) with the background radioactivity corrected. The collection of human tissue samples was approved by the ethical committee of Helsinki University Hospital, Finland.

### Immunofluorescence staining

Frozen 8-µm-thick tissue sections (*n* = 4) were fixed in 4% paraformaldehyde solution for 10 min and washed with PBS (pH 7.4) for 10 min. Sections were permeabilized with 0.3% Triton X-100 in PBS for 5 min and blocked for 1 h with 0.03% Triton X-100 in PBS containing 1% bovine serum albumin. Sections were incubated overnight at 4 °C with primary antibodies followed by 10 min washing with PBS. The sections were incubated with the corresponding fluorescent secondary antibodies for 2 h at 37 °C, counterstained with 2-(4-amidinophenyl)-1H-indole-6-carboxamidine (DAPI, Vectashield, Vector Laboratories, Newark, USA) to visualize the nuclei, and coverslips were mounted with Mowiol® (Sigma-Aldrich). The slides were scanned with a digital slide scanner (Pannoramic P1000, 3DHistech Ltd., Budapest, Hungary), and the images were viewed with Slideviewer (3DHistech Ltd., Budapest, Hungary).

### Stability of [^18^F]FNA-***S***-ACooP in rat plasma in vitro

The stability of [^18^F]FNA-*S*-ACooP was assessed in vitro using plasma from a Sprague Dawley rat. After incubating [^18^F]FNA-*S*-ACooP (0.2 MBq) in plasma (1.0 mL) at 37 °C for 5 min, 10 min, and 15 min, respectively, 50 μL samples were taken and plasma proteins precipitated with 50 µL acetonitrile. The mixture was then centrifuged, and the supernatant was injected into a reversed-phase C18 column for HPLC analysis at a flow rate of 5 mL/min, under radioactivity detection and UV detection. The HPLC solvent A was 0.1% TFA in water and solvent B was 0.1%TFA in acetonitrile. The elution gradient was from 10 to 50% B during 0–15 min. The experiments were performed in triplicates at each time point. The presence of intact [^18^F]FNA-*S*-ACooP in the plasma samples was confirmed with the reference standard of [^18^F]FNA-*S*-ACooP, and the presence of [^18^F]FNA as one of the radiometabolites was confirmed with the reference standard of [^18^F]FNA. [^18^F]FNA was prepared by hydrolyzing [^18^F]**1** with 1 M NaOH followed by neutralization with 1 M HCl and phosphate buffer (0.1 M) to pH 6–7.

## Results

### Preparation of 1 and FNA-*S*-ACooP

**1** was prepared by an esterification reaction in the presence of EDC as the coupling agent and a catalytic quantity of DMAP. Upon acid work up and flash column purification, the product was obtained in solid form in 49% yield. The chemical structure of **1** was confirmed by NMR (Additional file 1: Fig. S1) and HRMS. FNA-*S*-ACooP was synthesized by using **1** as the acylation agent in the presence of ACooP peptide in a one-pot reaction at r.t. To isolate the product, a semi-preparative HPLC was performed and the factions were collected. After removing the solvents, FNA-*S*-ACooP was obtained as a solid in 89% yield.

### Structural characterization of FNA-*S*-ACooP in comparison with FNA-*N*-ACooP

The chemical structure of FNA-*S*-ACooP was characterized by LC–MS and NMR analyzes, and the corresponding *N*-acylated compound FNA-*N*-ACooP was used as a reference. In the LC–MS/MS analysis (Additional file 1: Fig. S2), FNA-*S*-ACooP m/z [M + H]^1+^ was 969.4612 (theoretical value 969.4622) and the retention time was 11.05 min. To assign the MS fragments, the raw data were searched against the peptide sequence, including the modifications, using Proteome Discoverer 3.0 connected to a Mascot search engine. The Mascot search engine found the FNA moiety attached to the sulfhydryl group at the cysteine side chain instead of amino group at alanine-1 (Ala1). The fragmentations that proved the attachment site were m/z 397.13 (CGL y and a fragmentations), 484.17 (CGLS y and b fragmentations), 513.19 and 541.19 (CGLSG y and a/b fragmentations) and 626.28 and 654.27 (CGLSGL y and a/b fragmentations). In the NMR analysis, the individual amino acid residues were recognized mainly from the 2D total correlation spectroscopy (TOCSY) and heteronuclear single quantum coherence (HSQC) spectra, and further confirmed with 2D nuclear Overhauser effect spectroscopy (NOESY) and heteronuclear multiple bond correlation (HMBC) spectra (Additional file 1: Fig. S3–6). HMBC revealed the sequence based on the coupling of backbone amide protons with the adjacent carbonyl carbon. 1D ^1^H and ^13^C measurements were used for exact chemical shift assignments (Fig. 4, and Additional file 1: Fig. S7-S8). Cysteine protons were located at (in ppm) 3.37 and 3.51 (Cysβ), 4.65 (Cysα) and 8.86 (CysNH). Sulfhydryl proton was not found in the spectra. Alanine-1 proton peaks were broadened except Ala1β (in ppm): 1.34 (Ala1β), 3.89 (1 H, br, Ala1α)) and 8.05 (3 H, br, Ala1NH_3_^+^). Connections between Ala1 protons and FNA carbonyl carbon was missing but instead Cysβ protons were coupled to it on the HMBC spectrum. FNA carbonyl carbon was quite downfield at 188.07, which fits well to a thioester.

Next, we performed structural analyzes of custom-synthesized *N*-acylated compound FNA-*N*-ACooP by LC–MS and NMR (Fig. 4 and Additional file 1: Fig. S9-S15) as comparison with FNA-*S*-ACooP, and we indeed observed clear differences. FNA-*N*-ACooP m/z [M + H]^+1^ was 969.4617 which was identical as FNA-*S*-ACooP, but retention time was 13.03 min in comparison to that of FNA-*S*-ACooP at 11.05 min. The fragment ion spectra of m/z 969.46 looked different between the two samples. The spectrum of FNA-*N*-ACooP was simpler, and there were differences in fragment ions of the compounds. The Mascot search engine found the FNA moiety attached to Ala1 in FNA-*N*-ACooP. The fragmentations that proved the attachment site were m/z 246.13 (CGL y and a fragmentations), 588.28 (CGLSGLG y and b fragmentations) and 687.35 (CGLSGLGV y and b fragmentations). Compared to FNA-*S*-ACooP, in ^1^H NMR of FNA-*N*-ACooP, all cysteine protons were more upfield (in ppm): 2.75 and 2.81 (Cysβ), 3.31 (CysSH), 4.39 (Cysα) and 8.17 (CysNH) and Alanine-1 protons downfield (not Ala1β): 1.35 (Ala1β), 4.54 (Ala1α) and 8.84 (Ala1NH) for Ala1. Notably, Ala1 had only one NH proton located downfield from that of free NH_3_^+^ of unconjugated ACooP (8.04 ppm). Ala1NH and Ala1α protons were coupled to the carbonyl carbon of FNA (163.76 ppm) in the HMBC spectrum (Additional file 1: Fig. S15). Based on these data, the structure of compound FNA-*N*-ACooP was indeed *N*-acylated peptide.

### Radiosynthesis and identification of [^18^F]FNA-***S***-ACooP

To prepare [^18^F]**1**, we decided to test the on-resin ^18^F-fluorination method. Accordingly, [^18^F]fluoride was produced by an ^18^O(p,n)^18^F nuclear reaction using a cyclotron and extracted onto a PS-HCO_3_^−^ anion-exchange cartridge (Fig. 2). The cartridge was dried by passing through dry CH_3_CN followed by nitrogen gas. A mixture of precursor **4** and DABCO in CH_3_CN/tert-BuOH was pushed through the cartridge, and we indeed observed [^18^F]**1** as the only radioactive product. In this method, it was essential to pass the precursor solution slowly so that the precursor had more time to react with the [^18^F]fluoride bound on the cartridge. The ^18^F-fluorination efficiency had a positive correlation with the amount of precursor **4** (Additional file 1: Table S1). With 8 mg and 12 mg of compound **4**, 45% and 47% of [^18^F]**1** was formed in the presence of DABCO. When the amount of **4** was decreased to 2 mg and 4 mg, 9% and 23% of [^18^F]**1** was formed, respectively. Subsequently, [^18^F]**1** was purified with HPLC equipped with a semi-preparative C18 column and extracted onto two connected tC18 cartridges. [^18^F]**1** was eluted from the tC18 cartridges with CH_3_CN and ACooP peptide in borate buffer was added. In the presence of ACooP at concentrations of 1 mM, 3 mM and 5 mM, the conjugation efficiency at 10 min was 88% ± 2 (*n* = 3), 97% ± 3 (*n* = 3) and 97% ± 4 (*n* = 3), respectively (Additional file 1: Fig. S16). Conjugation time of 5 min was also applicable. Prolonged reaction time (e.g. 15 min) was not beneficial as side products started to form. After 10 min incubation at r.t., the reaction mixture was subjected to semi-preparative HPLC purification. Finally, [^18^F]**1** was eluted from the tC18 cartridge and formulated in PBS. The decay-corrected radiochemical yield was 29.9% ± 2.3 (*n* = 4) and radiochemical purity was 96.6% ± 2.3. The molar activity was 36.2 ± 22.0 MBq/nmol at the end of synthesis. The total synthesis time was 181.5 ± 6.9 min from the end of bombardment. The stability of [^18^F]FNA-*S*-ACooP in the injectable formulation (PBS with 14 mM ascorbic acid) was at least 4 h at r.t. and a longer shelf-life was not measured. Log*D*_7.4_ of [^18^F]FNA-*S*-ACooP was − 0.55 ± 0.01 (*n* = 3) indicating that it is a hydrophilic compound. The identity of [^18^F]FNA-*S*-ACooP was confirmed with HPLC analysis by using both FNA-*S*-ACooP and FNA-*N*-ACooP as references (Fig. 3).

### In vitro tissue binding

**Fig. 5:**
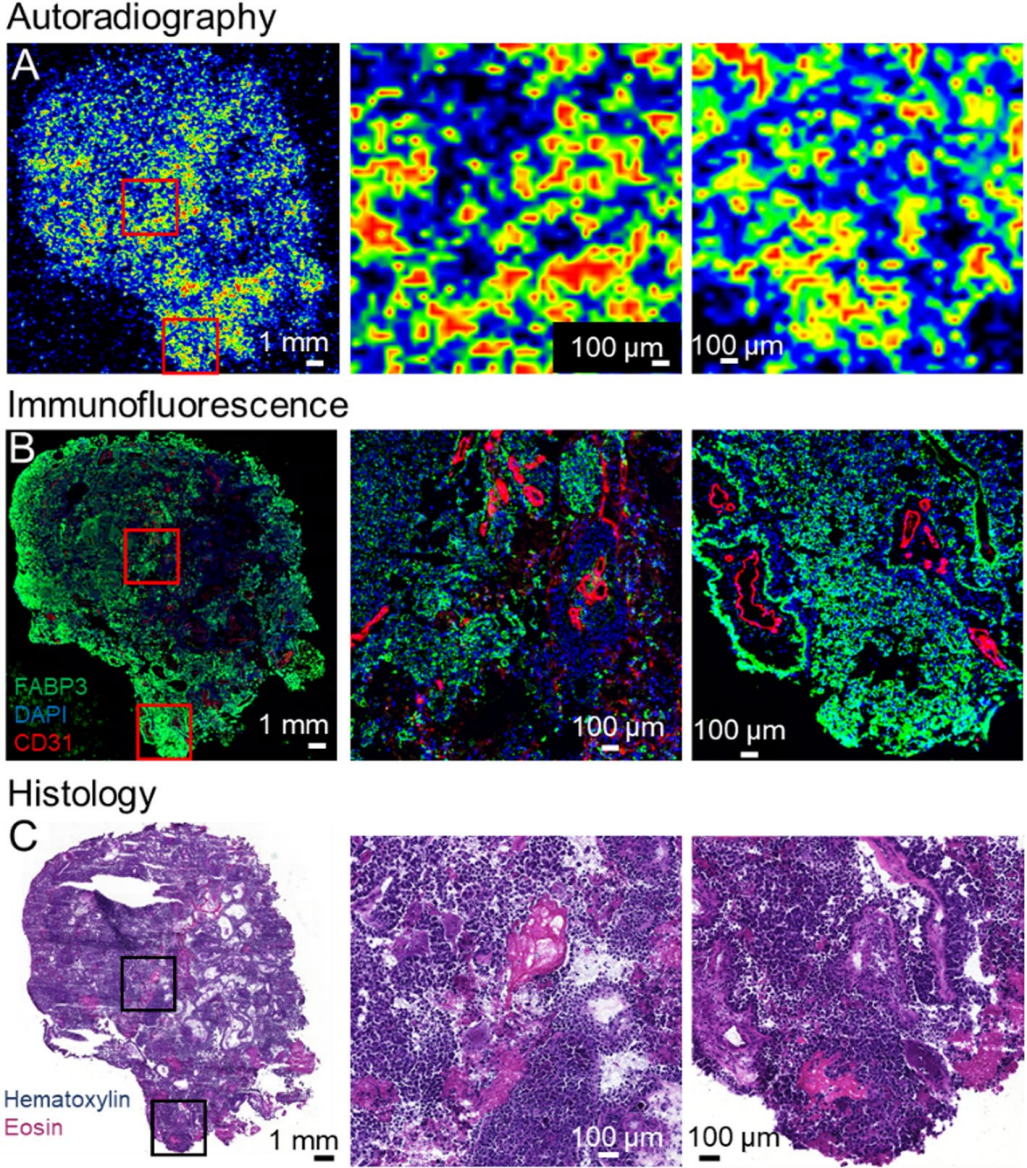
In vitro binding studies of [^18^F]FNA-*S*-ACooP in brain metastasis tissue sections from a patient with lung cancer. **A** Autoradiography, **B** immunofluorescence, **C** histology of adjacent sections. Left panels: whole tissue section; middle and right panels: high-power views of the areas within the red or black rectangles

Fresh brain metastasis tissue samples were collected from a patient with lung cancer, and after snap freezing, the samples were cryosectioned to 20 µm and 8 µm thick sections. The 20-µm sections were incubated in a solution of [^18^F]FNA-*S*-ACooP in PBS, rinsed with cold PBS and cold water, air dried briefly, and radioactivity was detected by autoradiography (Fig. 5A). Subsequently, the adjacent 8-µm sections were stained using antibodies against FABP3 and CD31 (biomarker for blood endothelial cells (Fig. 5B) and hematoxylin and eosin (H&E) (Fig. 5C). Autoradiography revealed heterogeneous and high focal [^18^F]FNA-*S*-ACooP binding in brain metastasis tissue sections from a patient with lung cancer, and the radioactivity binding mainly co-localized with the anti-FABP3 positivity detected in immunofluorescence staining of adjacent tissue sections (Fig. 5). To further confirm the binding specificity, blocking experiments were performed using native ACooP peptide as a blocker, and the [^18^F]FNA-*S*-ACooP binding was significantly reduced (*P* < 0.001, Additional file 1: Fig. S17). Radioactivity binding in the blocking experiments was 0.62 ± 0.09 PSL-background/mm^2^ (*n* = 4), which was 7.5-fold lower compared to the total binding of 4.64 ± 0.82 PSL-background/mm^2^ (*n* = 4) in the unblocked experiments.

### In vitro stability in rat plasma

The HPLC analysis showed that the percentage of intact [^18^F]FNA-*S*-ACooP remaining after incubating in the rat plasma in vitro for 5 min, 10 min, and 15 min was 8.2% ± 2.1, 2.9% ± 0.7, and 1.6% ± 0.5 (*n* = 3), respectively (Additional file 1: Fig. S18). In all the samples, [^18^F]FNA was observed as one of the radiometabolites and its amount was below 10% of the total radioactivity in the plasma samples. The chemical identity of the rest of the radiometabolites were not studied. These results indicated that [^18^F]FNA-*S*-ACooP was rapidly metabolized/degraded in rat plasma, but the thioester bond between the prosthetic group and the peptide was much more stable in comparison to peptide sequence itself (Additional file 1: Fig. S18).

## Discussion

In this work, we studied the radiolabeling of peptide ACooP with the activated ester of nicotinic acid [^18^F]**1** as the prosthetic compound (Fig. 2). In the chemical structure of ACooP, there is a free amino group and thiol group at the *N*-terminus, which provides opportunities for radiolabeling in different ways. We expected to conjugate ACooP with [^18^F]**1** by *N*-acylation, in a similar manner as reported in the literature about biomolecule radiolabeling with activated esters of nicotinic acid (Basuli et al. 2016; Basuli et al. 2020; Haskali et al. 2020; Keam 2021; Zhou et al. 2019). Interestingly, we observed that [^18^F]**1** conjugates with ACooP by *S*-acylation instead of *N*-acylation in a highly chemoselective manner, affording the formation of [^18^F]FNA-*S*-ACooP. To confirm the identity of the [^18^F]FNA-*S*-ACooP, the corresponding non-radioactive compound FNA-*S*-ACooP was prepared as a reference, and detailed characterization was performed with NMR and LC–MS analyzes. In addition, the *N*-acylated compound FNA-*N*-ACooP was analyzed as a reference for comparative study.

The prosthetic compound [^18^F]**1** was conveniently prepared by on-resin ^18^F-fluorination at r.t. without any need for azeotropic drying of [^18^F]fluoride. The efficiency of ^18^F-fluorination was increased along with the increasing amount of input precursor compound **4** according to our tests (Additional file 1: Table S1), which was in consistence with similar radiolabeling reactions reported previously (Basuli et al. 2016). This type of ^18^F-fluorination is based on aromatic nucleophilic substitution, and the reaction efficiency can be enhanced with additives including DABCO at 100–140 mM (Naumiec et al. 2017). In our experiments, the use of DABCO has increased the radiolabeling efficiency of [^18^F]**1** to a limited extent and it is not essential to use DABCO. The conjugation of [^18^F]**1** with ACooP was efficient under mild reaction conditions (Additional file 1: Fig. S16). Even at a concentration of 1 mM of ACooP, 88% of conjugation efficiency was achieved at 10 min of reaction. At higher concentrations (e.g. 3 mM and 5 mM) of ACooP, a reaction time of 5 min was sufficient. When the reaction time was prolonged to 15 min, side products started to form. For quality control, the end product was analyzed with HPLC equipped with a radioactivity detector. Unfortunately, multiple peaks were observed in analytical HPLC chromatograms in the initial quality control process. We have previously observed similar problems in the radiosynthesis tests of other radiopharmaceuticals, and radiolysis has turned out to be the reason (Li et al. 2015; Silvola et al. 2018). Therefore, we tested radiolysis prevention using ascorbic acid, propylene glycol or gentisic acid, of which ascorbic acid proved to be the most effective. In the presence of 14 mM ascorbic acid in PBS at pH 5–7, [^18^F]FNA-*S*-ACooP was stable for at least 4 h in the formulation solution.

To confirm the chemical identity of the radiolabeled end product, we purchased the non-radioactive *N*-acylated reference as a custom synthesis from United Biosystems, USA, because at the beginning we expected that end product was *N*-acylated compound. Accordingly, a sample of the end product was mixed with FNA-*N*-ACooP for co-injection for HPLC analysis. The retention times of these two peaks were indeed close to each other. However, they appeared in the wrong order in the HPLC chromatograms, with the FNA-*N*-ACooP peak appearing after the radiolabeled end product peak (Fig. 3A, B). In our HPLC system, the UV detector was placed before the radioactivity detector and the UV peak should precede the radioactivity peak, i.e. FNA-*N*-ACooP should appear slightly before and mostly overlap with the radiolabeled end product. This prompted us to prepare a non-radioactive sample under similar reaction conditions as used for the radiosynthesis for the chemical structure identification. Accordingly, we prepared **1** with an esterification reaction of 6- fluoronicotinic acid in the presence of 4-nitrophenol. The conjugation reaction of ACooP and FNA was carried out in a mixture of borate buffer (pH 8.6) and acetonitrile (70% by volume). The product was isolated with HPLC and dried under vacuum, and identified to be the *S*-acylated product FNA-*S*-ACooP. In the HPLC analysis with a sample of the radiolabeled product spiked with FNA-*S*-ACooP, the peaks occurred at nearly the same retention time as expected (Fig. 3A, C). Thus, it was plausible that in the radiosynthesis, the prosthetic compound [^18^F]**1** formed a thioester [^18^F]FNA-*S*-ACooP with cysteine instead of an amide with alanine at the amino terminus. The *S*-acylation was highly chemoselective and only trace amount of *N*-acylated product [^18^F]FNA-*N*-ACooP was observed under the used conjugation conditions (borate buffer, pH 8.6). In the identification process of [^18^F]FNA-*S*-ACooP, both FNA-*S*-ACooP and FNA-*N*-ACooP were used as references. They were characterized in detail with NMR and LC–MS analyzes (see Results).

Commercial FNA-*N*-ACooP was prepared by solid-phase peptide synthesis, and the amino group at the *N*-terminus was acylated with 6-fluoronicotinic acid while the side chain of the cysteine residue was still protected. In our radiosynthesis, [^18^F]**1** was conjugated to ACooP bearing deprotected side chains and the free thiol group at the cysteine residue was available for the acylation reaction. Unexpectedly, in the radiolabeling of ACooP, thioester formation was exclusive and only < 1% of *N*-acylation was observed. In the absence of thiol group as a competing nucleophile, [^18^F]**1** could indeed react with amino group under certain reaction conditions, for example in the presence of triethylamine as a base in DMSO as a solvent (Haskali 2020). Accordingly, we studied ACooP labeling with [^18^F]**1** in DMSO containing trimethylamine at different temperatures, but similar results were observed.

Having [^18^F]FNA-*S*-ACooP produced with high radiochemical purity, we proceeded to in vitro tissue binding studies as the first step in the PET application development process. The in vitro experiments were performed with brain metastasis tissue sections from a patient with lung cancer that we have detected to express the target FABP3 (Fig. 5). We stained the tissues with anti-FABP3 antibodies and we indeed confirmed abundant target expression. Autoradiography revealed heterogeneous and high focal [^18^F]FNA-*S*-ACooP binding, which mainly co-localized with the anti-FABP3 positivity (Fig. 5).H&E and anti-CD31 staining confirmed presence of viable tumor tissue. Complete co-localization was not anticipated when using serial tissue sections for autoradiography and immunostaining due to the heterogeneity in tumor samples. We then performed blocking experiments using molar excess of native ACooP peptide as a blocker. The binding was indeed reduced to large extent in compared to the total binding (*P* < 0.001, Additional file 1: Fig. S17). Thus, the target binding specificity was further confirmed. In addition, we conducted stability tests of [^18^F]FNA-*S*-ACooP in rat plasma and observed that it was metabolized quickly. After 5 min of incubation, only 8.2% of the intact [^18^F]FNA-*S*-ACooP remained. After 15 min of incubation, there was little to no intact [^18^F]FNA-*S*-ACooP observed (Additional file 1: Fig. S18). In all the samples, the amount of [^18^F]FNA was below 10%, indicating that the instability was primarily due to the peptide sequence itself rather than the thiolester bond. To further explore the potential use of [^18^F]FNA in radiolabeling biomolecules through *S*-acylation, it will be necessary to select biomolecules with higher stability than ACooP.

## Conclusion

We have prepared the prosthetic compound [^18^F]**1** conveniently with an on-resin ^18^F-fluorination method, and a new radiopharmaceutical [^18^F]FNA-*S*-ACooP. Highly chemoselective thioester bond formation was observed when using the [^18^F]**1** prosthetic group under the described labeling conditions for ACooP peptide labeling. 6-[^18^F]Fluoronicotinic acid is an emerging labeling moiety in the preparation of radiopharmaceuticals for clinical PET imaging, and this work contributes to the knowledge of using it as a prosthetic group for radiolabeling of biomolecules. FABP3-positivity in the human tissue samples indicates that FABP3 targeting has clinical relevance. In vitro studies with tissue sections showed that [^18^F]FNA-*S*-ACooP binding has partial correlation with FABP3-positivity, and targeting specificity was further confirmed with blocking experiments. Further preclinical studies are warranted to evaluate the potential of [^18^F]FNA-*S*-ACooP as a PET imaging agent.

### Supplementary Information

**Additional file 1.** Supplementary experimental data including NMR and MS spectra, HPLC chromatograms of in vitro plasma stability studies etc.

## Data Availability

Those data are provided in the Supporting Information.

## Abbreviations

ACooP: Peptide with sequence of H-ACGLSGLGVA-NH_2_
FABP3: Fatty acid binding protein 3
FNA: Fluoronicotinic acid
