# Supplementary material for: Radiosynthesis, structural identification and in vitro tissue binding study of [18F]FNA-S-ACooP, a novel radiopeptide for targeted PET imaging of fatty acid binding protein 3
Source: EJNMMI Radiopharm Chem. 2024 Feb 23;9:16. doi: 10.1186/s41181-024-00245-3 (PMC10891031; doi:10.1186/s41181-024-00245-3)
Supplement: Supplementary file 1 — Additional file 1. Supplementary experimental data including NMR and MS spectra, HPLC chromatograms of in vitro plasma stability studies etc. [file 41181_2024_245_MOESM1_ESM.docx]

***Supplementary information (SI)***

**Radiosynthesis, structural identification and *in vitro* tissue binding study of [^18^F]FNA-*S*-ACooP, a novel radiopeptide for targeted PET imaging of fatty acid binding protein 3**

Pyry Dillemuth^a‡^, Tuomas Karskela^a‡^, Abiodun Ayo,^b^ Jesse Ponkamo,^a^ Jonne Kunnas,^a,c^ Johan Rajander,^d^ Olli Tynninen,^e^ Anne Roivainen,^f,g,h^ Pirjo Laakkonen,^b,i,j^ Anu J. Airaksinen,^a^ Xiang-Guo Li^a,f,h*^

^a^Turku PET Centre and Department of Chemistry, University of Turku, Turku, Finland.

^b^Translational Cancer Medicine Research Program, Faculty of Medicine, University of Helsinki, Helsinki, Finland.

^c^Pharmaceutical Sciences Laboratory, Faculty of Sciences and Engineering, Åbo Akademi University, Turku, Finland.

^d^Accelerator Laboratory, Åbo Akademi University, Turku, Finland.

^e^Department of Pathology, Helsinki University Hospital and University of Helsinki, Helsinki, Finland.

^f^Turku PET Centre, University of Turku and Turku University Hospital, Turku, Finland.

^g^Turku Center for Disease Modeling, University of Turku, Turku, Finland.

^h^InFLAMES Research Flagship, University of Turku, Turku, Finland.

^i^Laboratory Animal Centre, HiLIFE University of Helsinki, Helsinki, Finland.

^j^iCAN Flagship Program, University of Helsinki, Helsinki, Finland.

^‡^ Pyry Dillemuth and Tuomas Karskela contributed equally to this work.

***Corresponding Author:** Assistant Professor Xiang-Guo Li, PhD, Turku PET Centre, Kiinamyllynkatu 4-8, FI-20520 Turku, Finland; Phone: +358504485069; E-mail: [xiali@utu.fi](mailto:xiali@utu.fi)

## 1. Methods for liquid chromatography-mass spectrometry (LC-MS) analysis

The LC-ESI-MS/MS analysis was performed on a nanoflow HPLC system (Easy-nLC1000, Thermo Fisher Scientific) coupled to the Q Exactive HF mass spectrometer (Thermo Fisher Scientific) equipped with a nano-electrospray ion source. Peptides were first loaded onto a trapping column (100 µm ID × 2 cm)* and subsequently separated inline on an analytical column (75 μm ID × 15 cm). The mobile phase consisted of water with 0.1% formic acid (solvent A) and acetonitrile:water (80:20, v/v) with 0.1% formic acid (solvent B). Peptides were eluted with the following gradient: from 13% to 55% of solvent B in 10 min, from 55% to 100% of solvent B in 5 min followed by washing for 5 min at 100% of solvent B. MS data was acquired automatically using Thermo Xcalibur 4.1 (Thermo Fisher Scientific). A data-dependent acquisition method consisted of repeated cycles of MS1 scan covering a range of m/z 300–2000 followed by HCD fragment ion scans (MS2 scans) for up to the 10 most intense precursor ions from the MS1 scan. Stepped collision energy (NCE 23%, NCE 25% and NCE 29%) was used in HCD fragmentation.

**2. ^18^F-fluorination efficiency tests in the preparation of [^18^F]1**

The efficiency of on-resin ^18^F-fluorination with varying amounts of compound **4** was examined by first trapping the [^18^F]fluoride (22.5-118 MBq) onto a Chromafix 30-PS-HCO_3_ anion-exchange cartridge (Macherey-Nagel GmbH & Co. KG, Düren, Germany). The cartridge was flushed with anhydrous acetonitrile and dried with N_2_ gas for 3 min. [^18^F]**1** was synthesized by passing the solution of compound **4** with or without 1,4-diazabicyclo[2.2.2]octane (DABCO) (14 mg, 0.13 mmol) in acetonitrile:tert-butanol (1:4, v/v, 1.2 mL) through the cartridge. The on-resin synthesis efficiency was tested with compound **4** in amounts of 2 mg, 4 mg, 8 mg, and 12 mg (Table S1). After the elution, the radioactivity remained on the 30-PS-HCO_3_-cartridge and the radioactivity of the product [^18^F]**1** were measured. The ^18^F-fluorination efficiency was increased by increasing the amount of precursor **4** from 2 mg up to 12 mg. When 8 mg and 12 mg of compound **4** were used, [^18^F]**1** was formed in 44.5% ± 2.9 and 47.3% ± 1.7, respectively (n = 3) (Table S1). In the presence of DABCO, relatively higher amount of [^18^F]**1** (Table S1, entry 3) was formed in comparison with the reaction in the presence of DABCO (Table S1, entry 4). However, we observed approximately 7% of side product in the presence of DABCO, and the amount of side product increased along with time after elution from the cartridge. Taken together, it is not essential to use DABCO in the radiosynthesis of [^18^F]**1**.

**Table S1** ^18^F-fluorination efficiency of [^18^F]**1**

| Entry | Amount of compound **4** (mg)* | Formation of [^18^F]**1** (%) |
| --- | --- | --- |
| 1 | 2 | 9.3 ± 2.6 (*n* = 3) |
| 2 | 4 | 23.0 ± 8.1(*n* = 3) |
| 3 | 8 | 44.5 ± 2.9 (*n* = 6) |
| 4 | 8** | 36.0 ± 5.1 (*n* = 3) |
| 5 | 12 | 47.3 ± 1.7 (*n* = 3) |

## *In the presence of 14 mg DABCO. **In the absence of DABCO3. NMR and HRMS spectra for 1


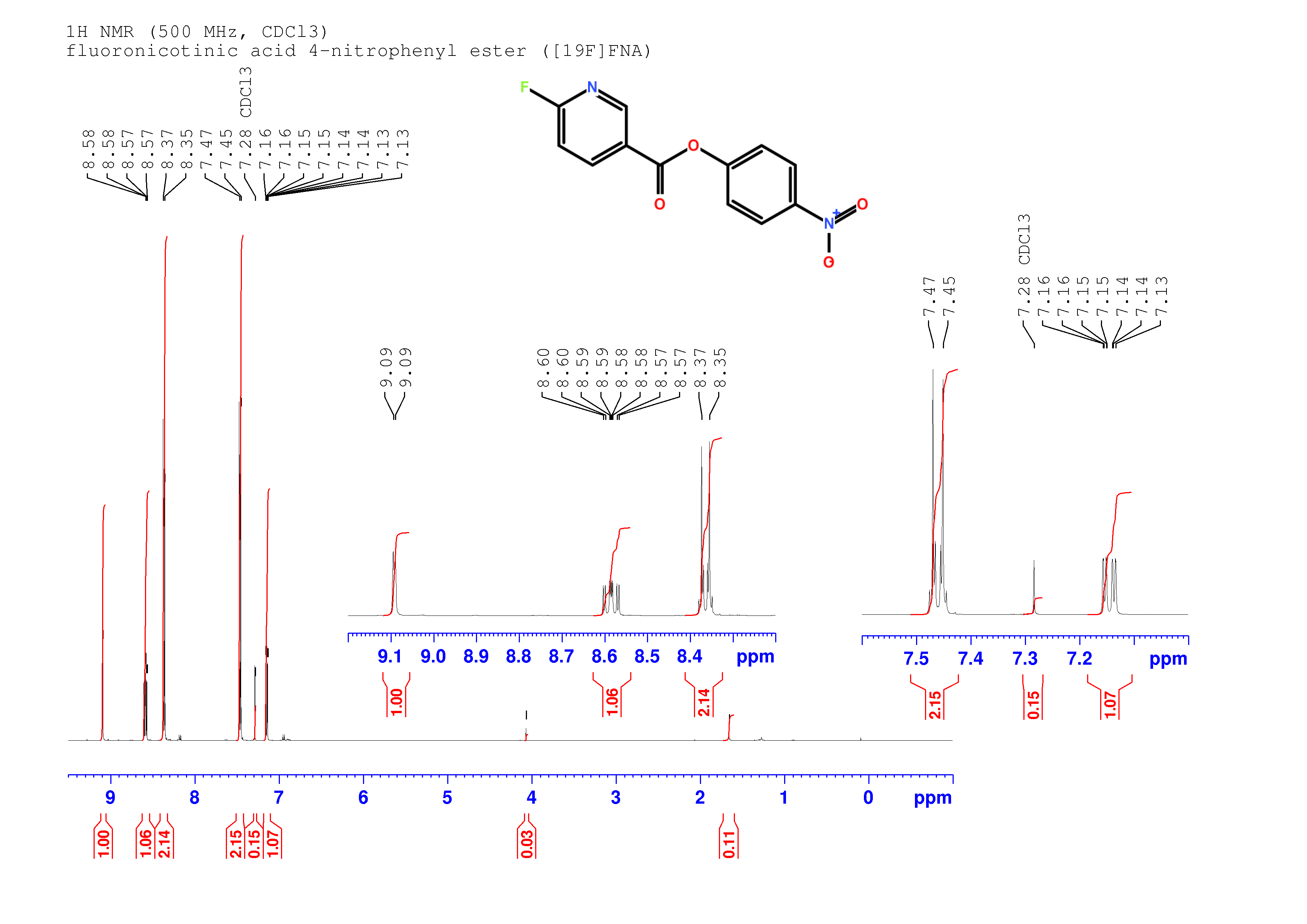


**
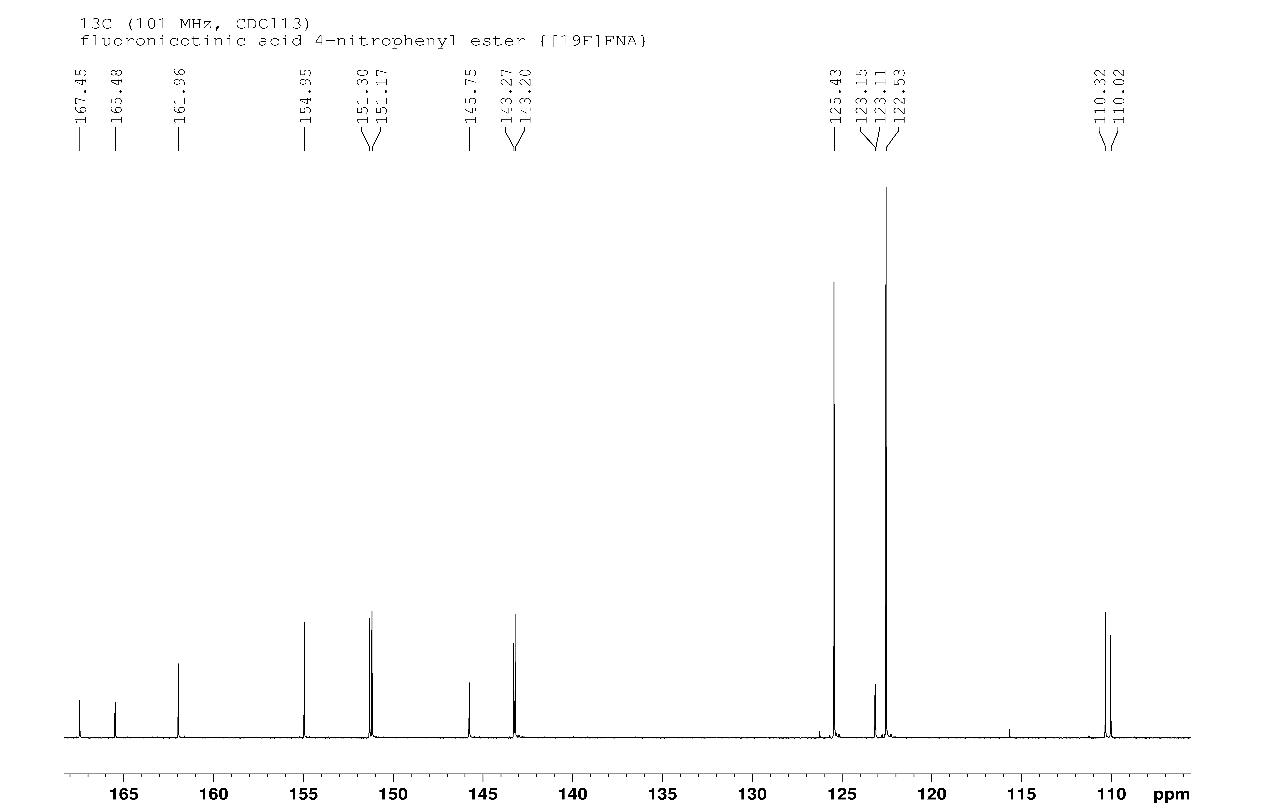
**

**
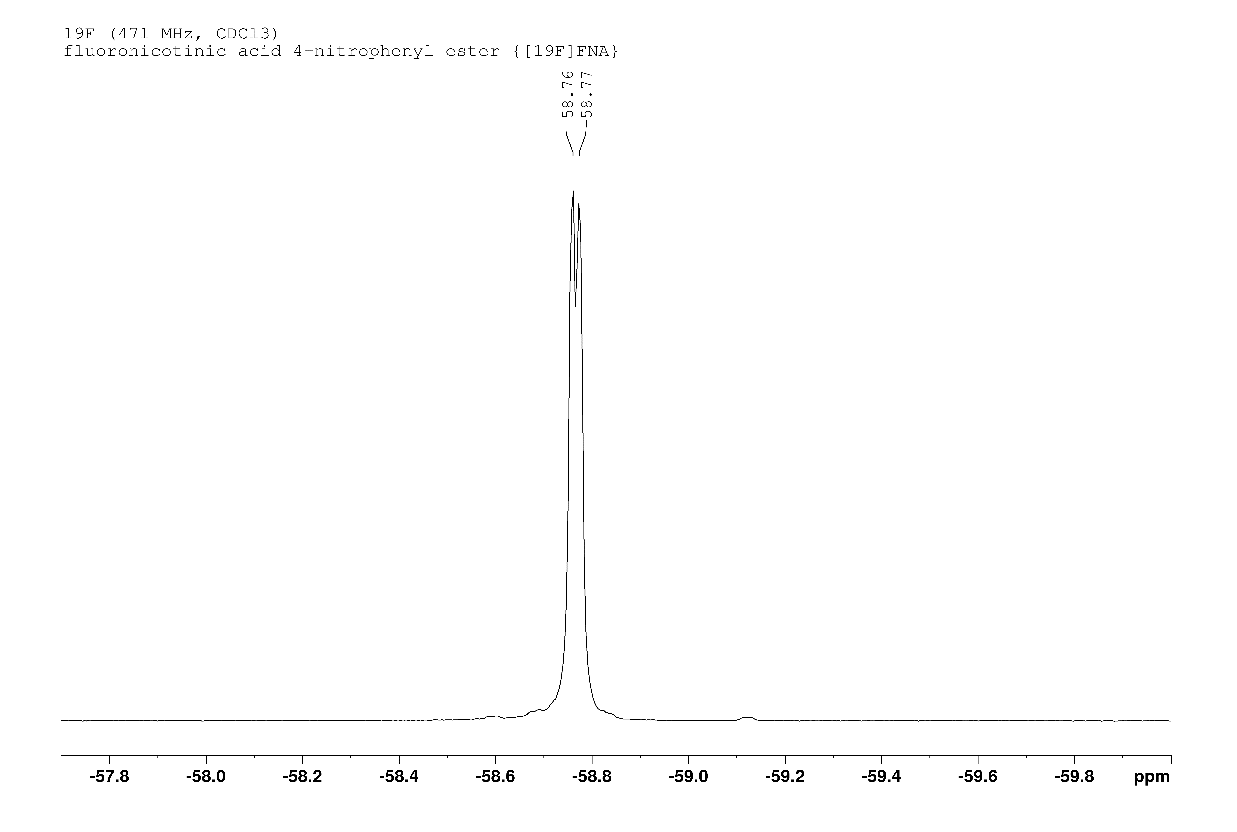
**

**Fig. S1** ^1^H-NMR, ^13^C-NMR, ^19^F-NMR and HRMS of **1**

## 4. MS and NMR spectra for FNA-*S*-ACooP





**Fig. S2** MS2 spectrum of precursor ion m/z 969.9626 of FNA-*S*-ACooP


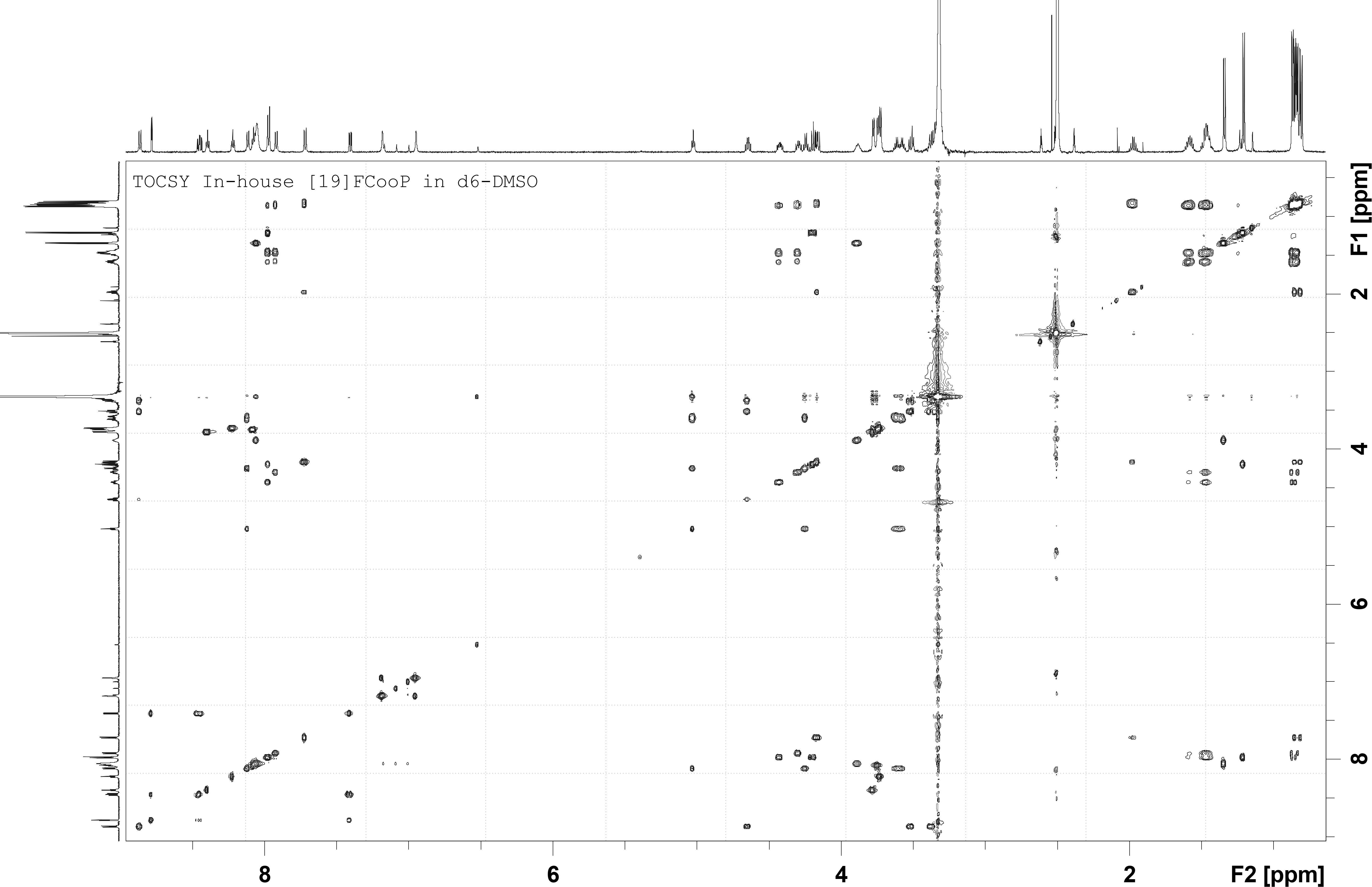


**Fig. S3** TOCSY NMR spectrum of in-house made FNA-*S*-ACooP


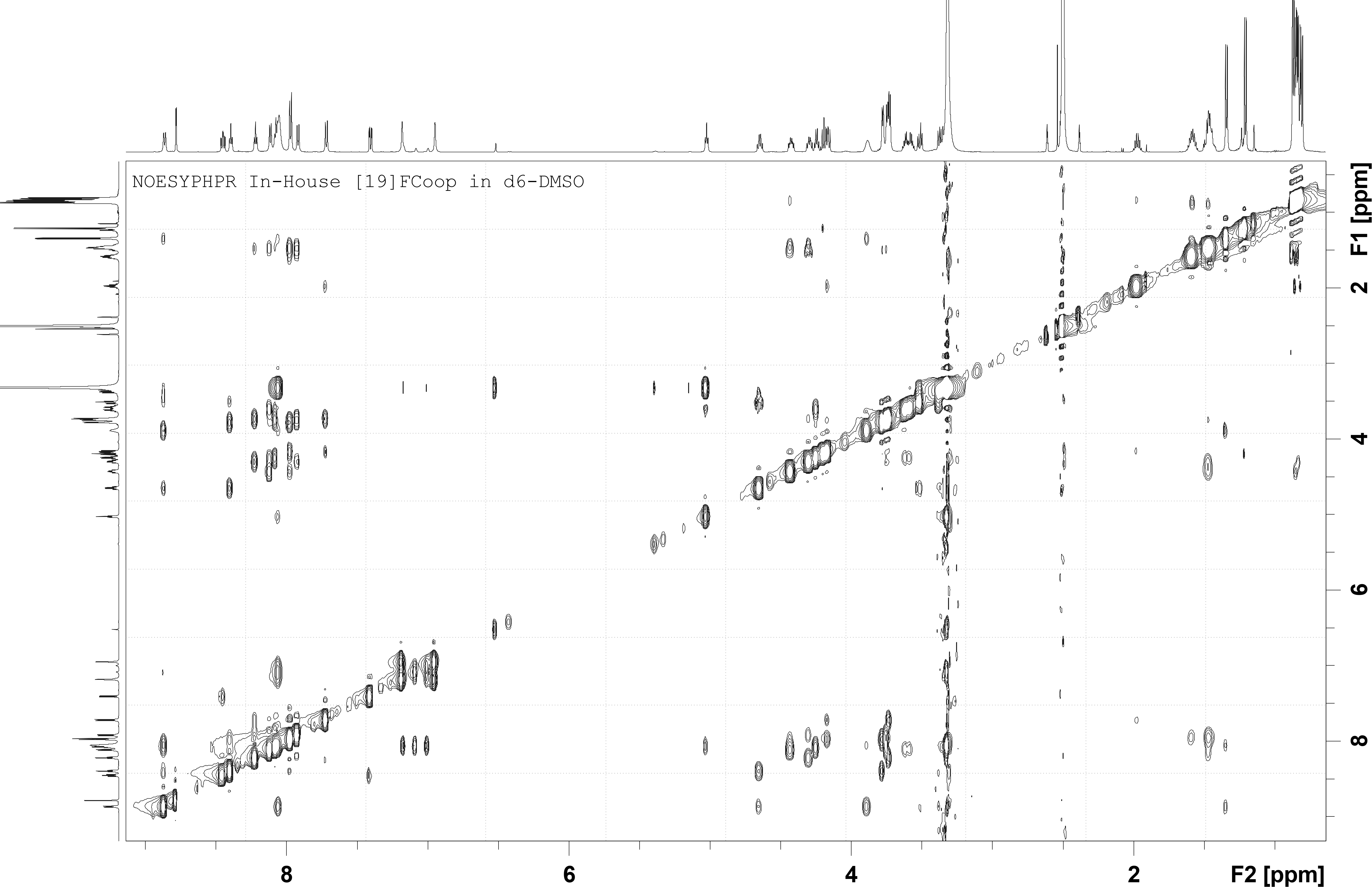


**Fig. S4** NOESY NMR spectrum of in-house made FNA-*S*-ACooP


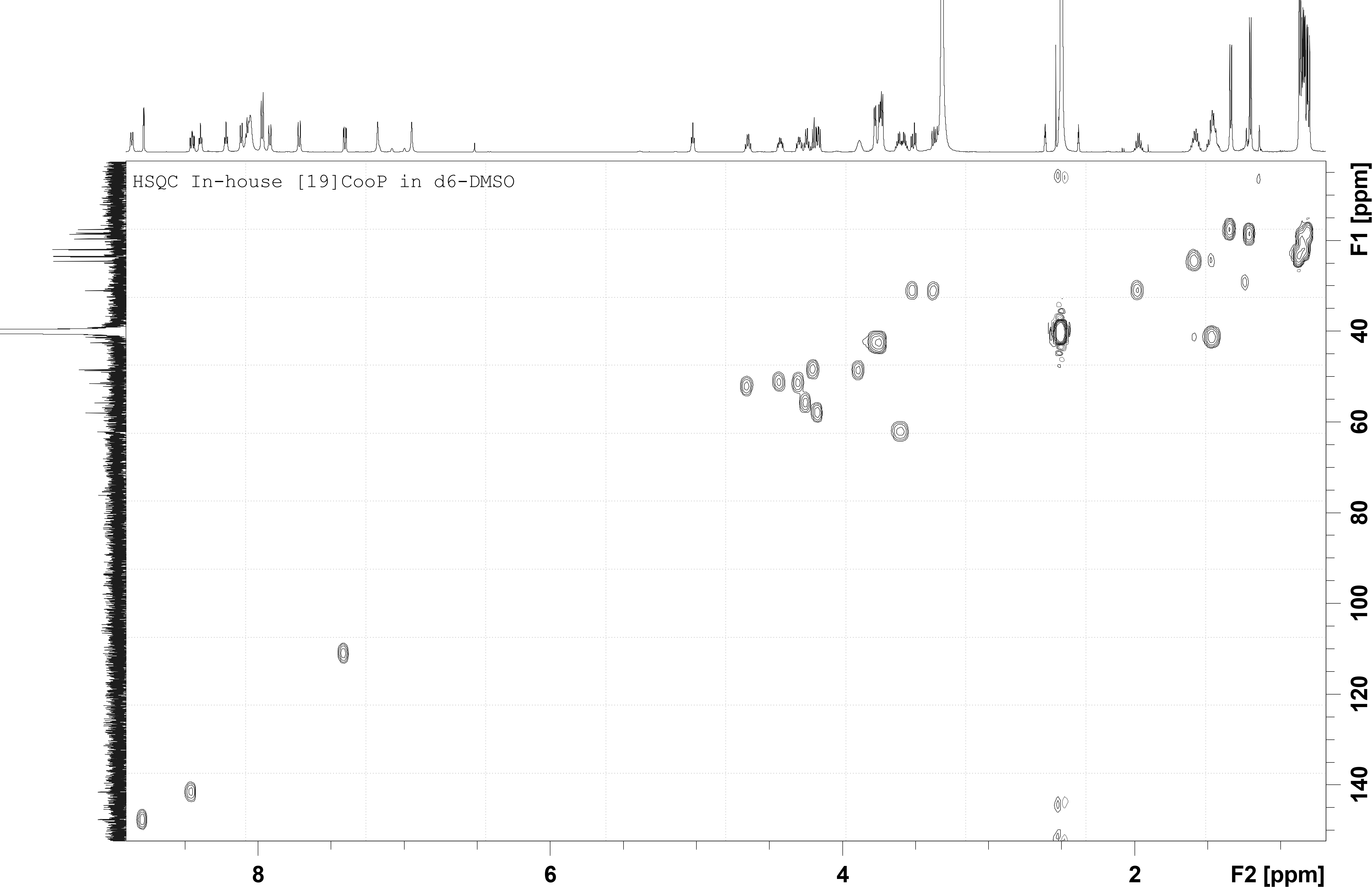


**Fig. S5** HSQC NMR spectrum of in-house made FNA-*S*-ACooP


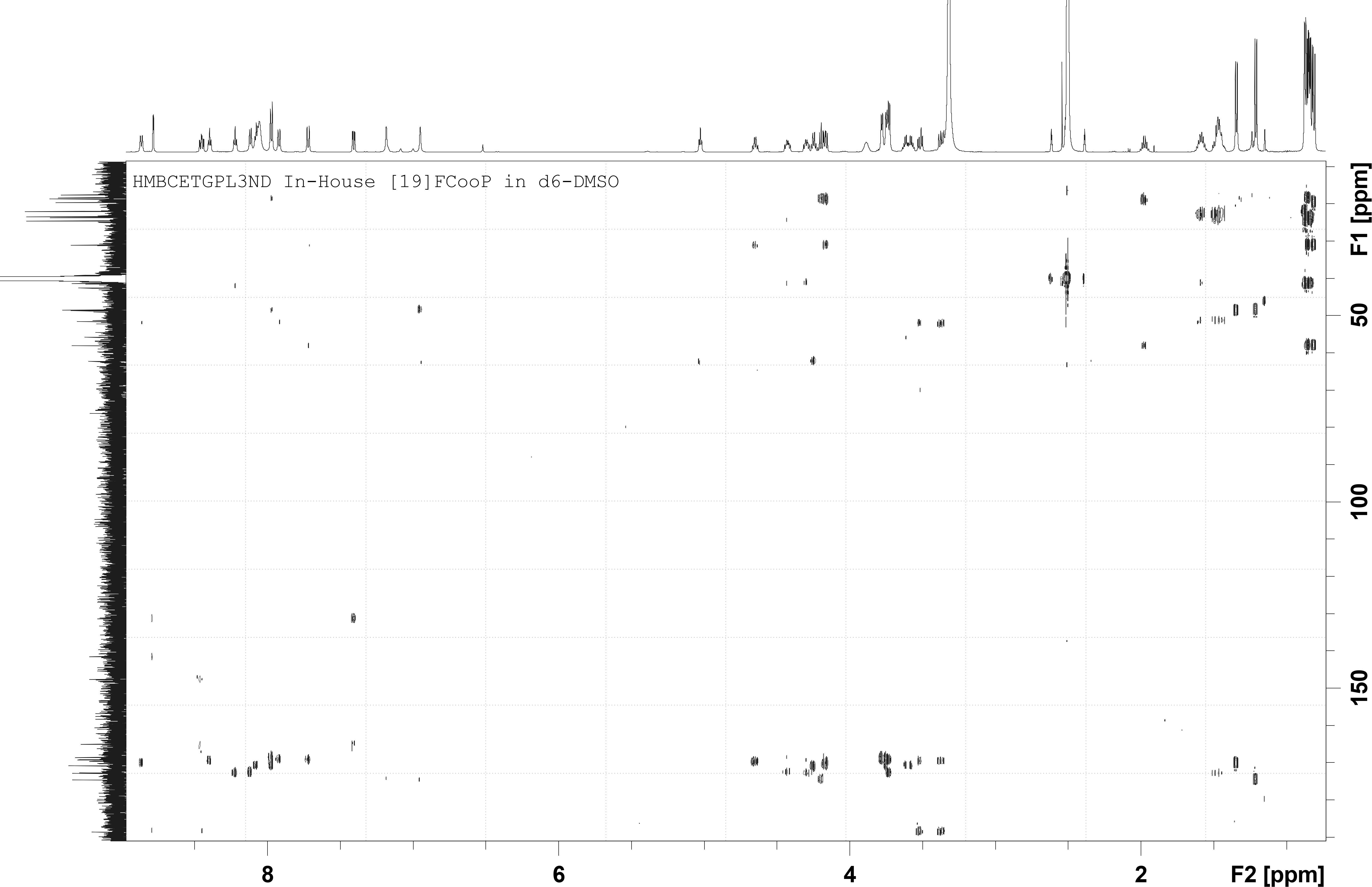


**Fig. S6** HMBC NMR spectrum of in-house made FNA-*S*-ACooP


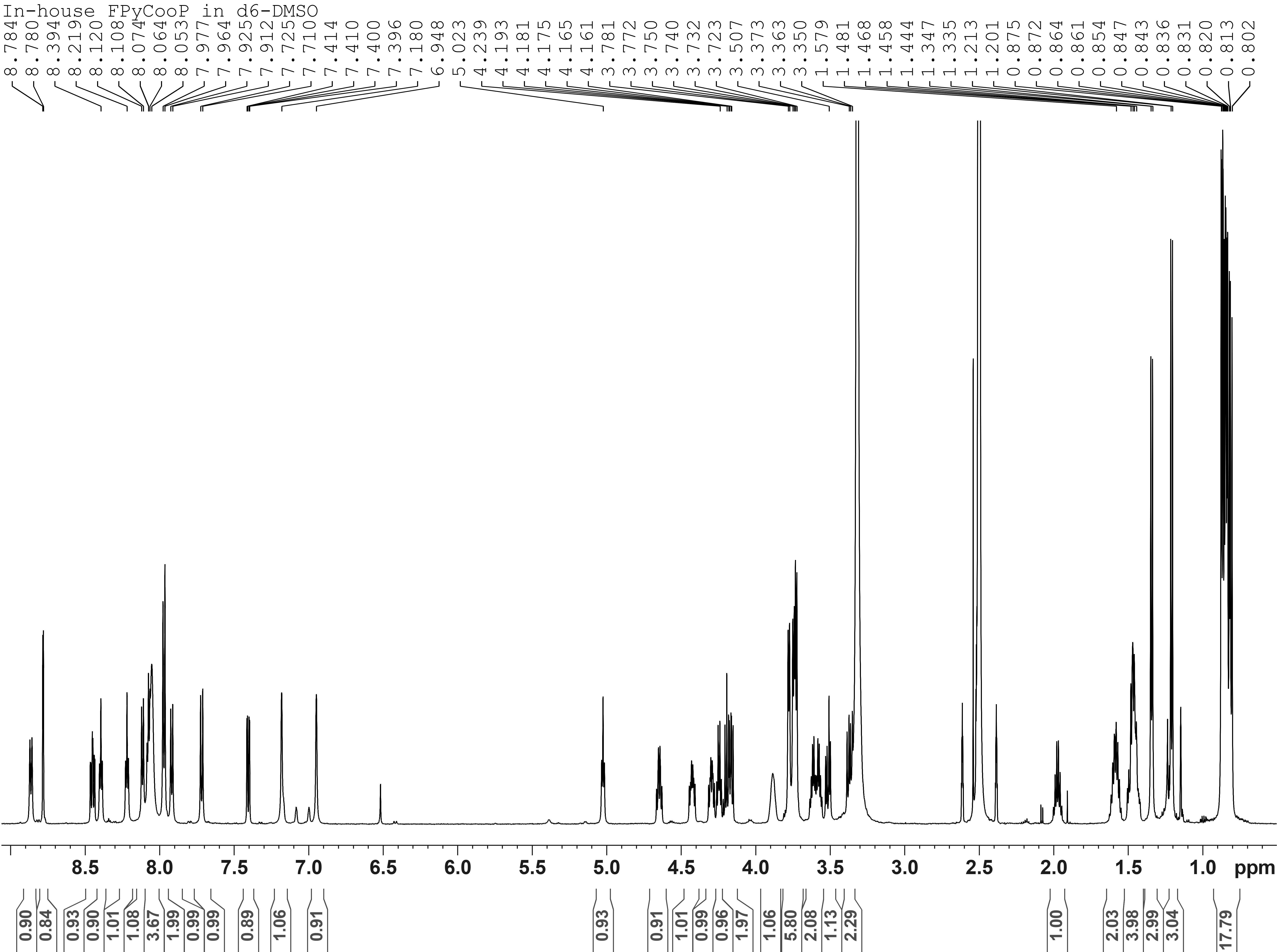


**Fig. S7** ^1^H NMR spectrum of in-house made FNA-*S*-ACooP


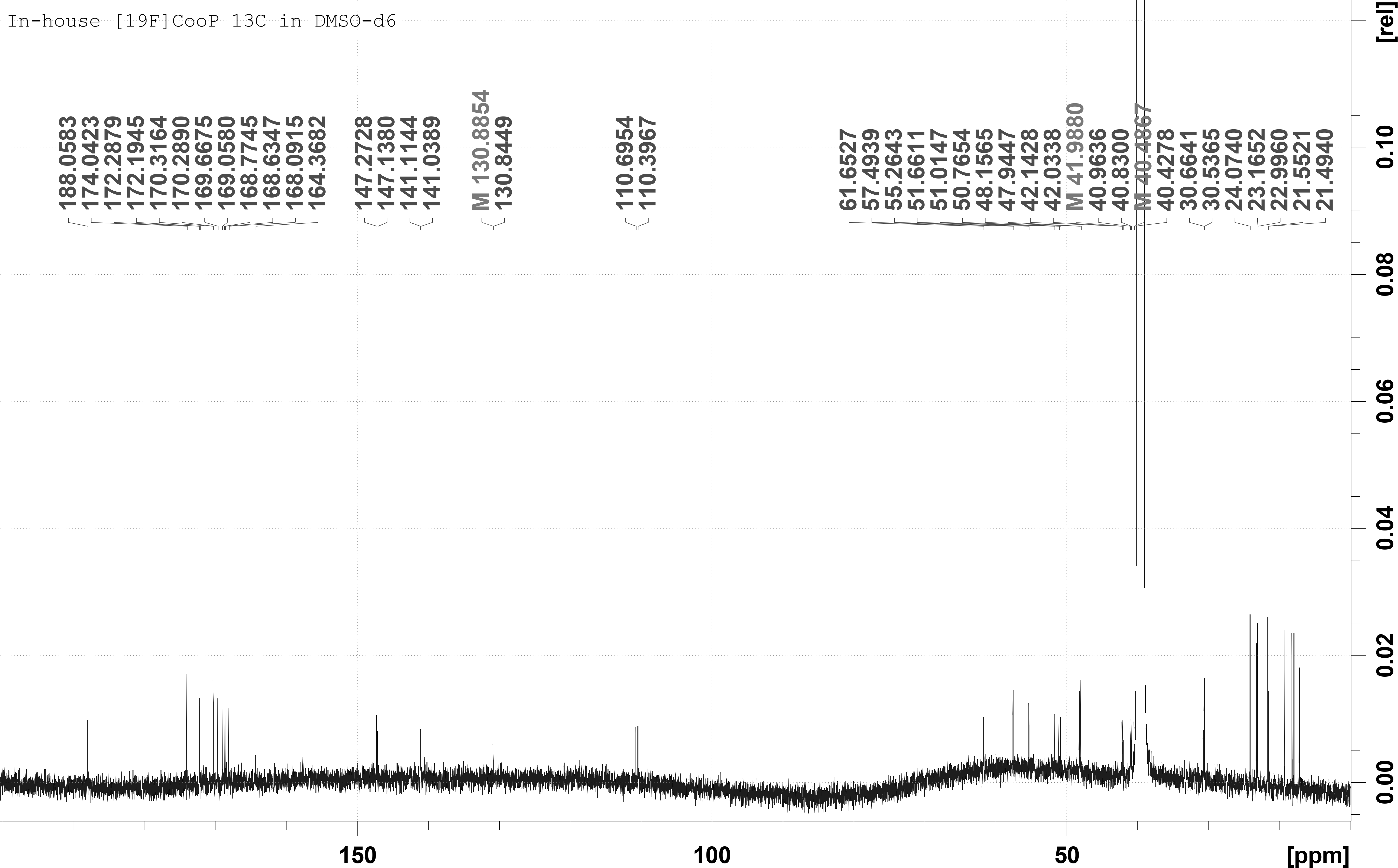


**Fig. S8** ^13^C NMR spectrum of in-house made FNA-*S*-ACooP

## 5. MS and NMR spectra of FNA-*N*-ACooP





**Fig. S9** MS2 spectrum of precursor ion m/z 969.4619 of reference compound FNA-*N*-ACooP


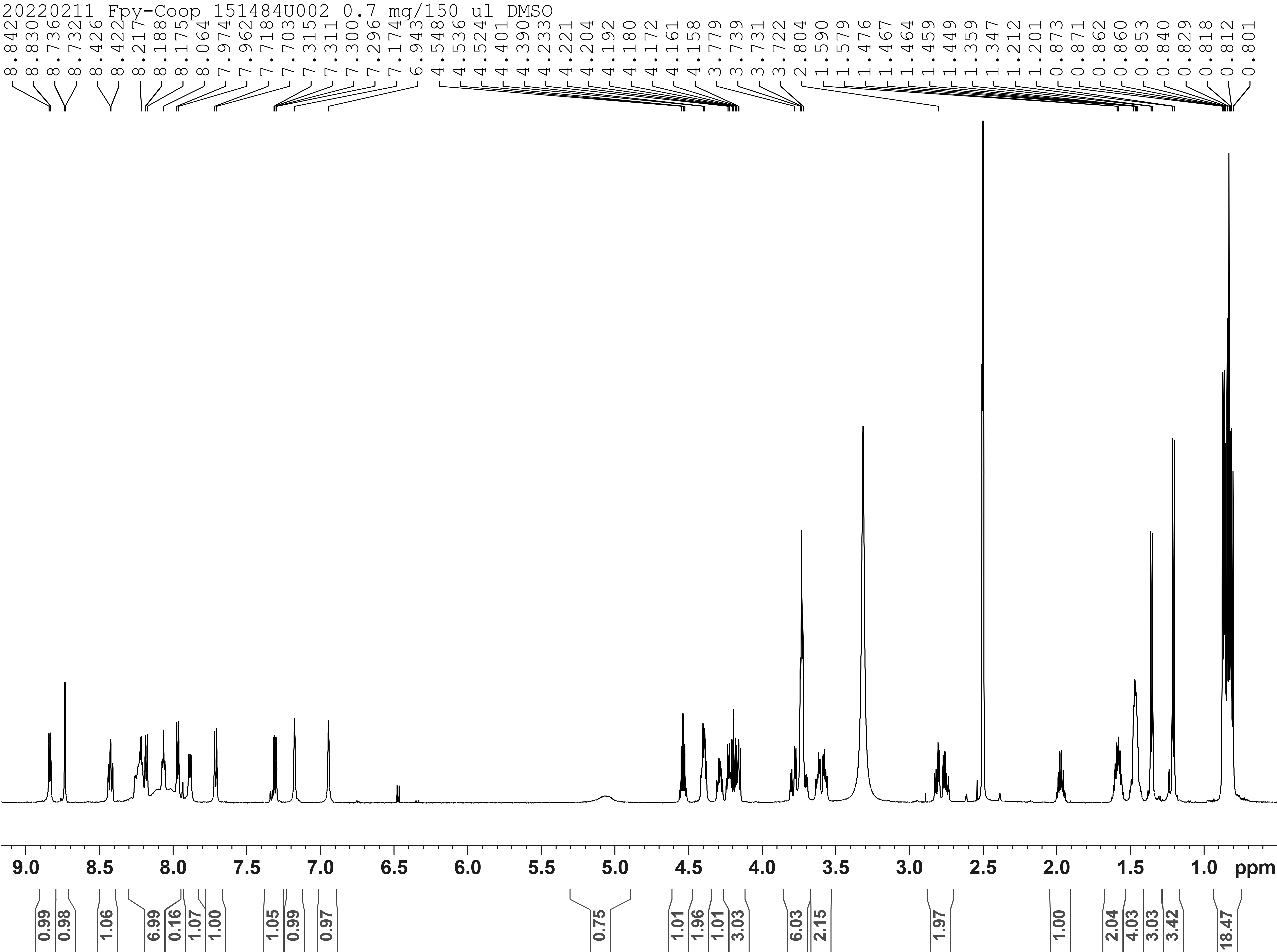


**Fig. S10** ^1^H NMR spectrum of reference compound FNA-*N*-ACooP


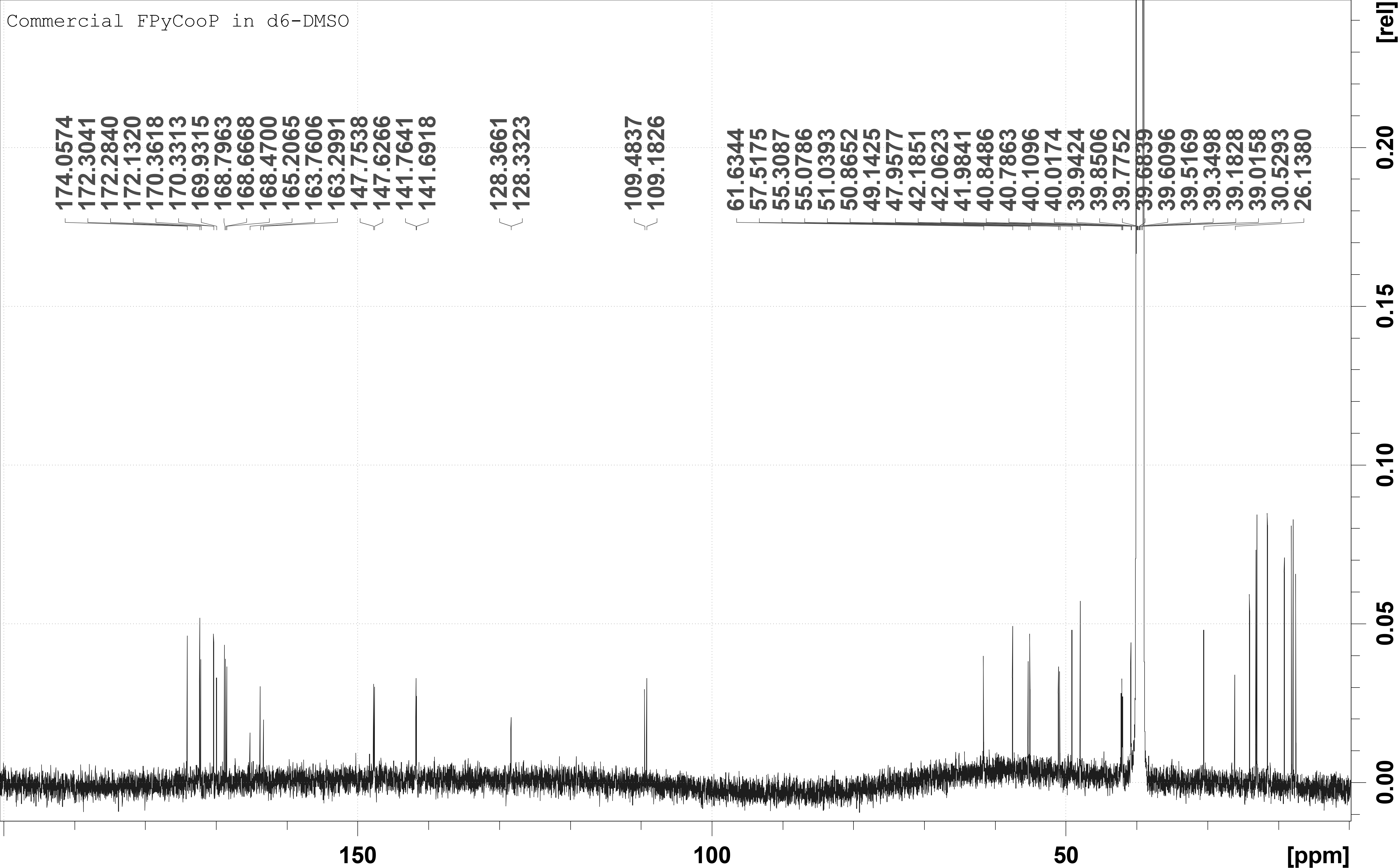


**Fig. S11** ^13^C NMR spectrum of reference compound FNA-*N*-ACooP


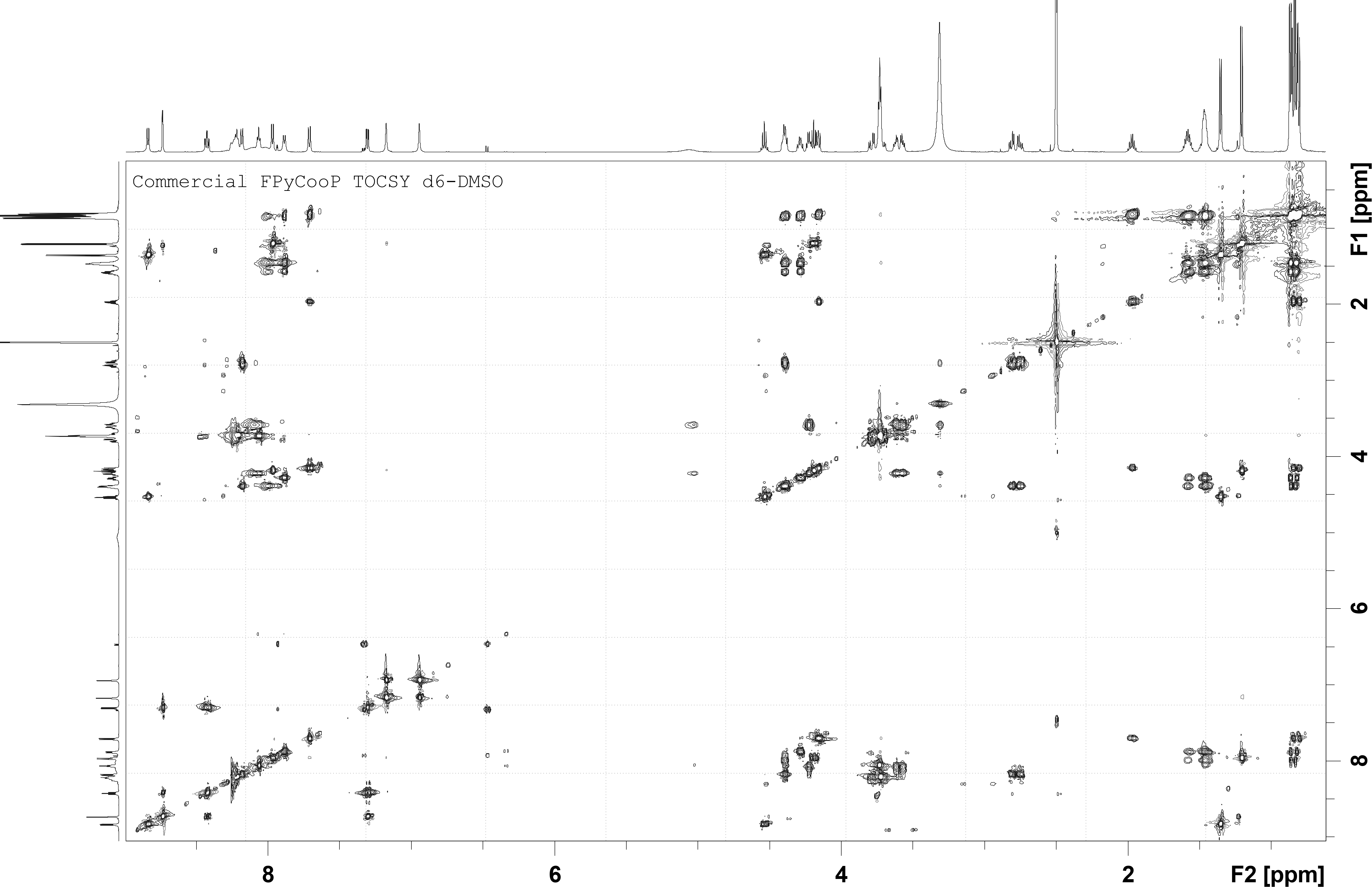


**Fig. S12** TOCSY spectrum of reference compound FNA-*N*-ACooP


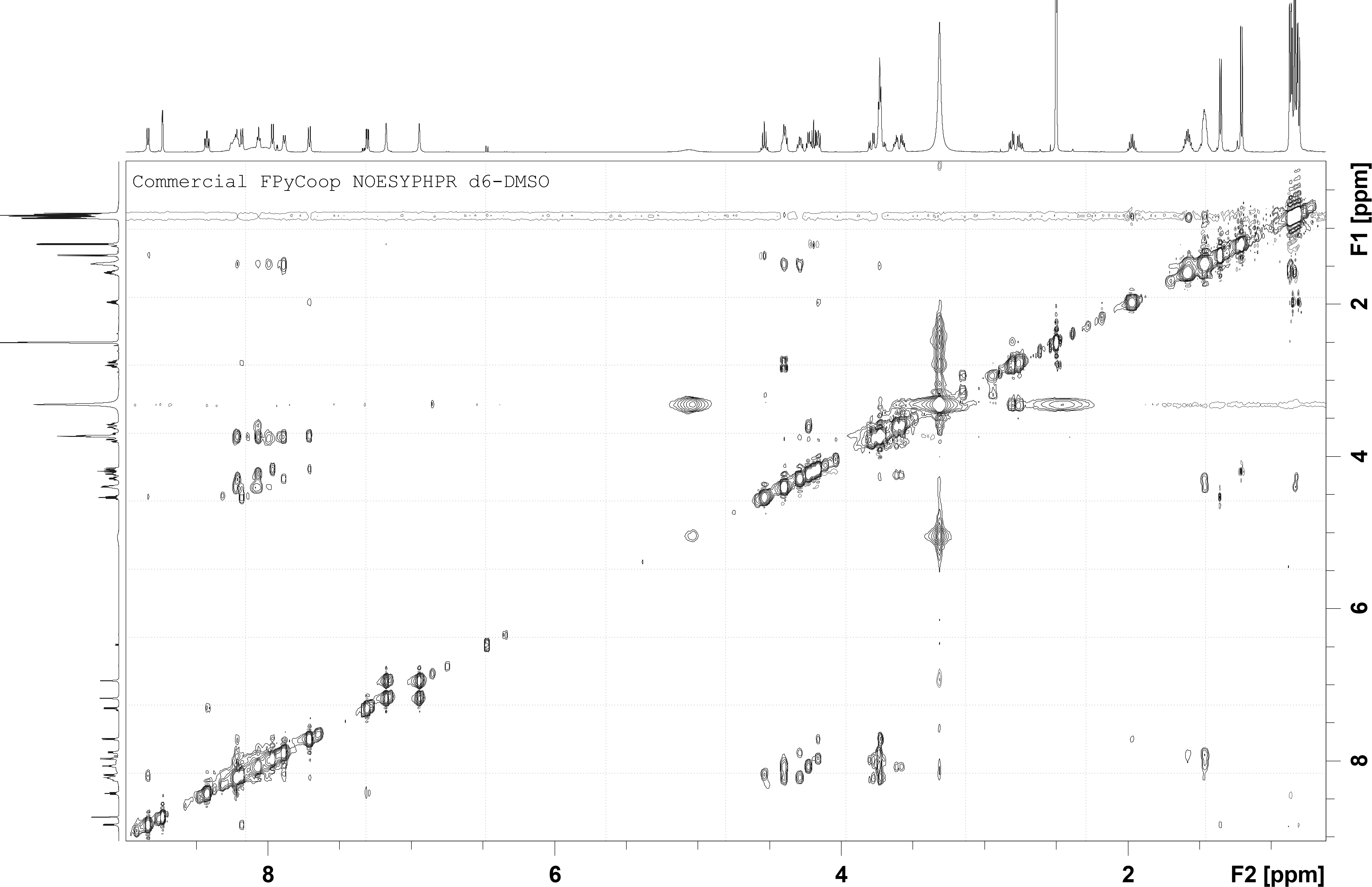


**Fig. S13** NOESY spectrum of reference compound FNA-*N*-ACooP


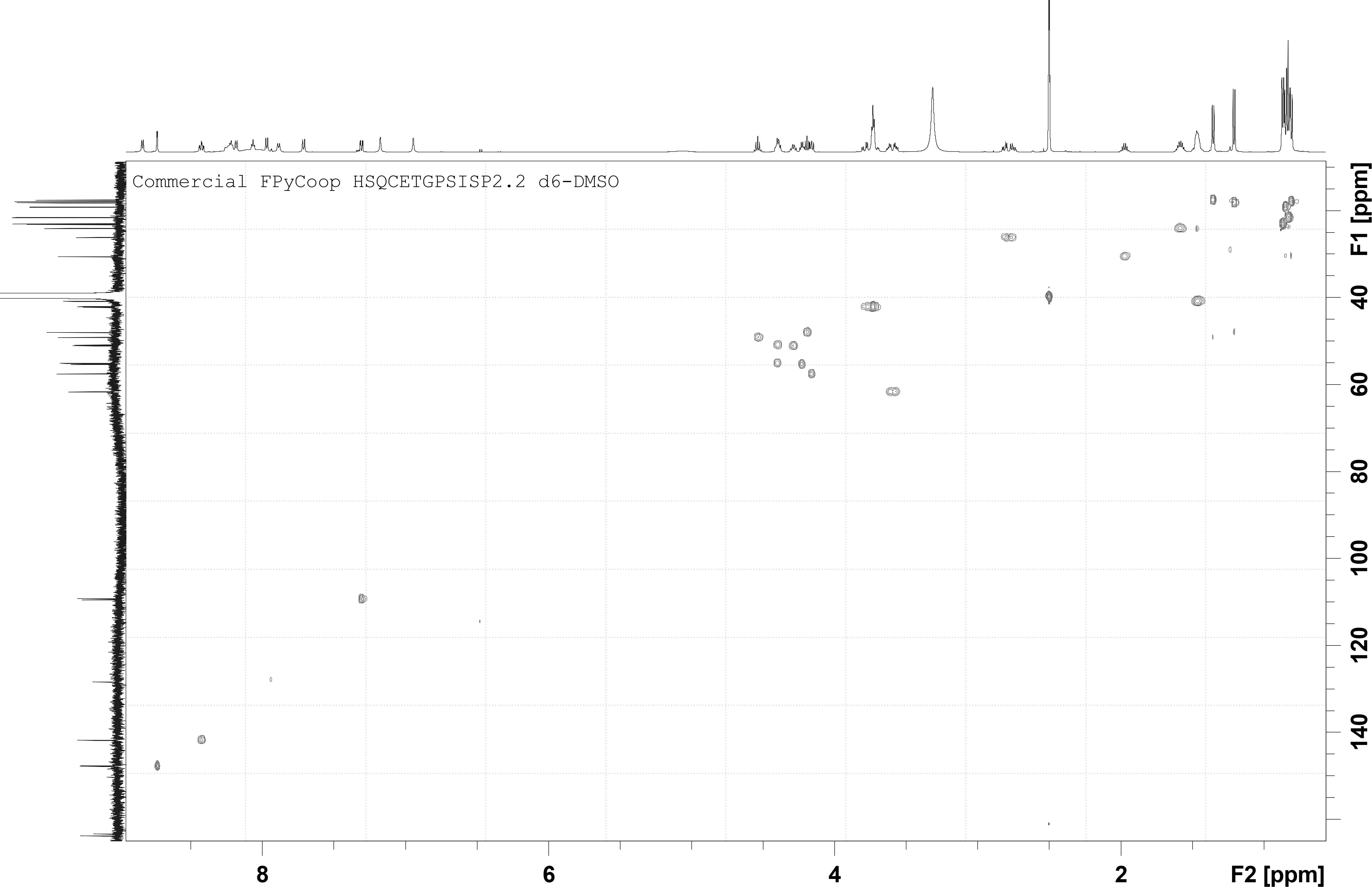


**Fig. S14** HSQC spectrum of reference compound FNA-*N*-ACooP


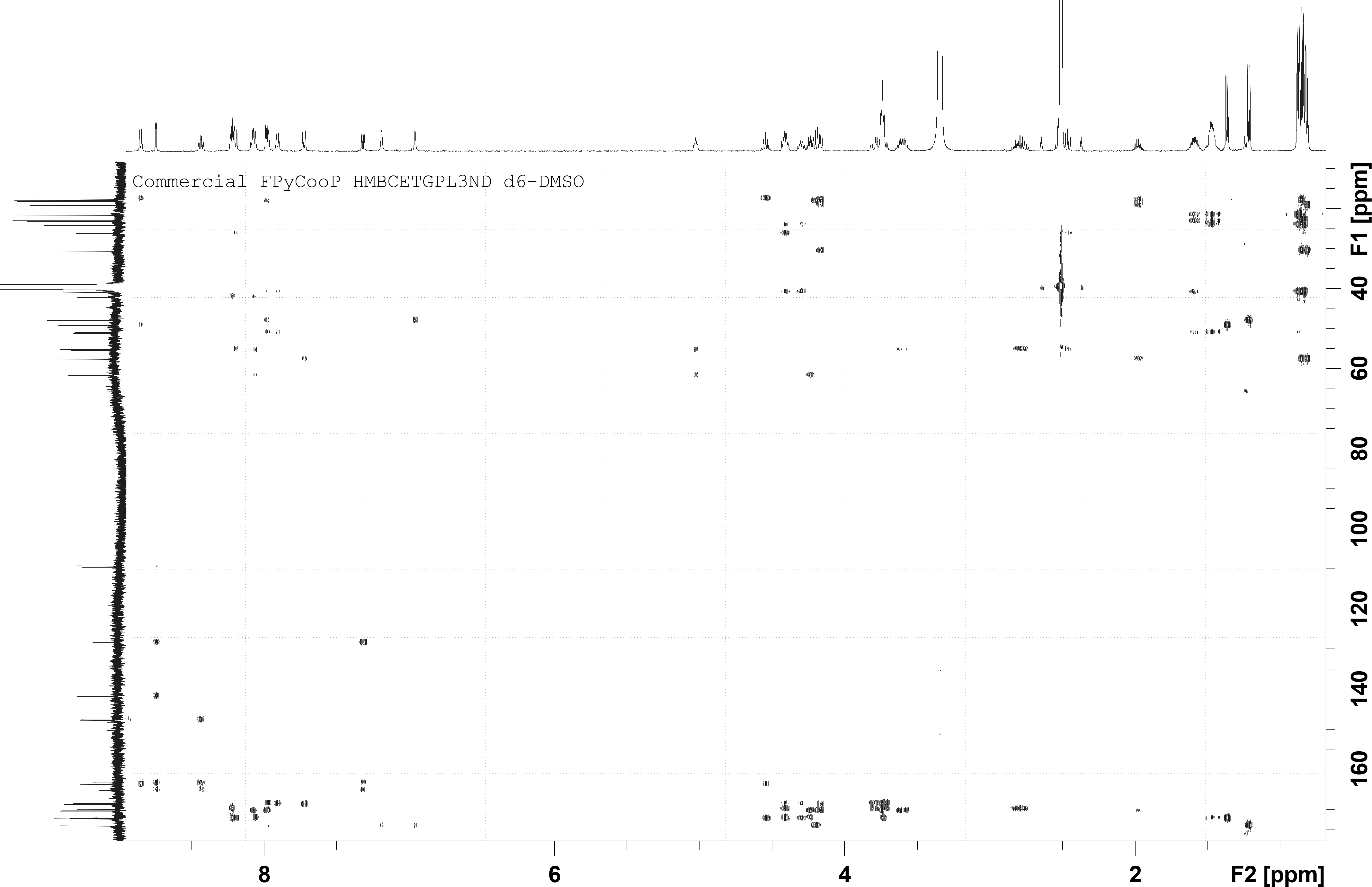


**Fig. S15** HMBC spectrum of reference compound FNA-*N*-ACooP

**6. ACooP conjugation efficiency tests**

[^18^F]**1** was incubated with ACooP at concentrations of 1 mM, 3 mM and 5 mM in borate buffer at pH 8.6 at r.t., respectively. Samples (1 μL each) were taken at time points of 1, 5, 10, 15 min, and immediately applied onto a silica gel 60 F₂₅₄ thin-layer chromatography (TLC) plate. Subsequently, the TLC plates were developed with a mobile phase of 95% acetonitrile in water, and then apposed to a BAS-TR2025 phosphor imaging plate and scanned with a BAS-1800II scanner. The percentage of peptide formation was quantified using TINA 2.0 software. At ACooP concentration of 5 mM for 10 min reaction, the conjugation efficiency was 97.3% ± 4.2 (*n* = 3, Fig. S16). At concentrations of 1 mM and 3 mM, the conjugation efficiency became lower. Regarding reaction time, the optimal range was 5─10 min. When the reaction time was prolonged to 15 min, side products started to appear.

**Fig. S16** Conjugation efficiency of [^18^F]**1** with ACooP at different concentrations and time points to prepare [^18^F]FNA-S-ACooP

**7. In vitro tissue blocking experiment**


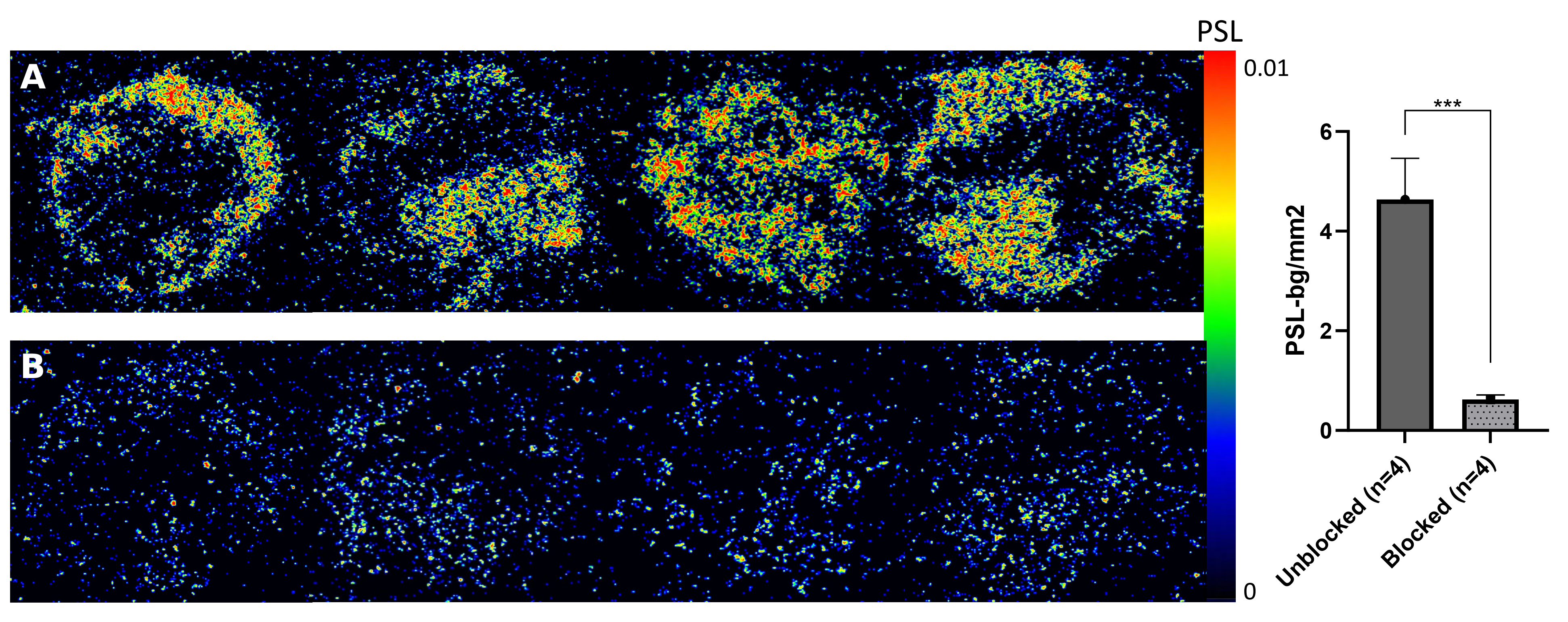


**Fig**. **S17** *In vitro* total binding and blocked binding in brain metastasis cryosections from a patient with lung cancer. A) Total binding in the presence of [^18^F]FNA-*S*-ACooP (0.016 MBq/mL) in phosphate-buffered saline. B) Blocking experiments of adjacent tissue sections with native ACooP peptide (5 µM) under similar conditions. The blocking effect was statistically significant (P <0.001).

**8. HPLC chromatograms for *in vitro* stability study of [^18^F]FNA-*S*-ACooP in rat plasma**

#
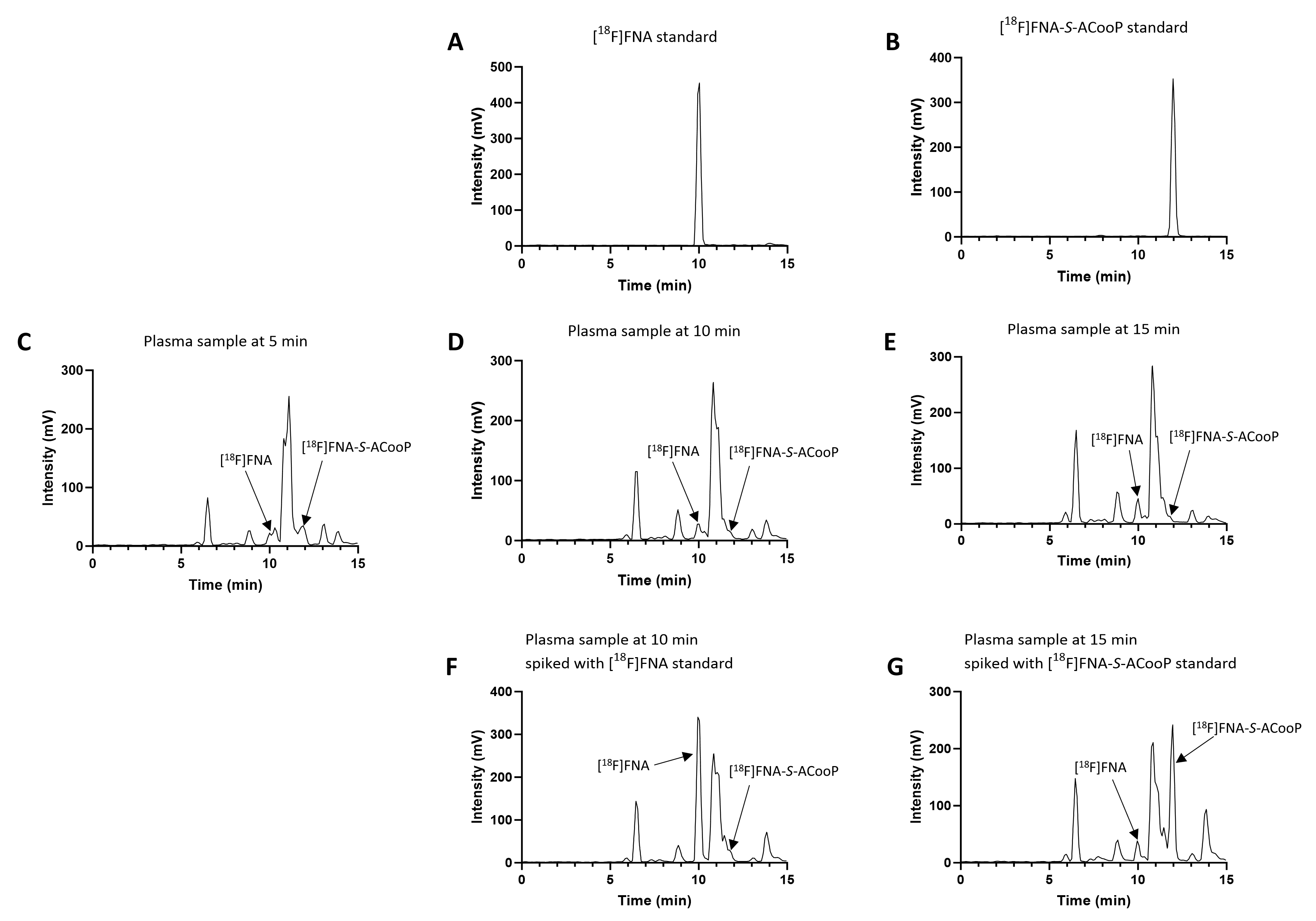
Fig. S18 *In vitro* stability of [^18^F]FNA-*S*-ACooP in rat plasma as analyzed by HPLC under radioactivity detection. A) The retention time of reference standard of [^18^F]FNA was 10.0 min. B) The retention time of reference standard of [^18^F]FNA-*S*-ACooP was 11.8 min. Representative chromatograms of plasma samples C) at 5 min, D) at 10 min, E) at 15 min, F) plasma sample at 10 min spiked with reference standard [^18^F]FNA, and G) plasma sample at 15 min spiked with reference standard [^18^F]FNA-*S*-ACooP.
